# Acid‐Catalyzed Rearrangement Reaction for Single‐Molecule Junction Formation

**DOI:** 10.1002/chem.71233

**Published:** 2026-06-06

**Authors:** Yihao Zhang, Yunlong Li, Zhenpin Lu, Haixing Li

**Affiliations:** ^1^ Department of Physics City University of Hong Kong Kowloon Hong Kong SAR China; ^2^ Department of Chemistry City University of Hong Kong Kowloon Hong Kong SAR China

**Keywords:** benzidine rearrangement reaction, deprotonation, hydrazobenzene, scanning tunneling microscope break‐junction, single‐molecule junction conductance

## Abstract

Single‐molecule junction formation is not possible for hydrazobenzenes due to the absence of anchoring groups for making contact with the Au electrodes. In stark contrast, with the addition of acid, the junction formation is immediate and robust, monitored in real‐time via the emergence of the 1.3×10^−3^ G_0_ conductance peak by the scanning tunneling microscope‐based break junction method. We propose that this single‐molecule junction is benzidine attaching to Au electrodes through NH_2_→Au dative interactions, which is a rearrangement reaction product of hydrazobenzene, supported by measurements of ex situ synthesized benzidine and high‐performance liquid chromatography analysis. Critically, we show that in forming the metal‐molecule‐metal junctions under an applied voltage in a nonpolar solvent, the reaction products possibly become deprotonated, which otherwise requires an addition of base. We find that for synthesized ‐NH_3_^+^ terminated compounds, ‐NH_3_^+^ is also possibly converted into ‐NH_2_ upon the junction formation. These results underscore a new acid‐catalyzed method for single‐molecule device formation.

## Graphical abstract

Hydrazobenzenes cannot form single‐molecule devices with gold electrodes in a nonpolar solvent, but with the addition of trifluoroacetic acid, robust single‐molecule junctions are created. Hydrazobenzenes likely undergo rearrangement reactions to form benzidines, which are then attached to gold contacts by dative NH_2_→Au interactions for junction formation.

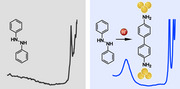

## 1 Introduction

Catalyzing or accelerating chemical reactions on solid–liquid interfaces is an emerging frontier in surface physics and chemistry. As an ideal platform to investigate the chemical reactions at the single‐molecule scale, the scanning tunneling microscope‐based break‐junction (STM‐BJ) technique not only provides a tunable electric field between the electrodes that may facilitate reactions, but also enables real‐time monitoring of chemical reactions through simultaneous measurements of single‐molecule conductance [1, 2, 3, 4, 5, 6, 7, 8, 9]. Several recent examples demonstrate that the oriented electric field or the Au surface in an STM‐BJ setup catalyzes chemical reactions. Albert C. Aragonès et al. [10] reported that the oriented electric field between an STM gold tip and a gold substrate can enhance the rate of a Diels–Alder reaction. It has also been reported that an acylation reaction exhibited significantly enhanced reaction rates on a Au surface, which were further increased upon an applied electric field [11]. Electric fields can facilitate electron transfer and stabilize polarized transition states, thereby enabling selective bond reorganization that is otherwise challenging to achieve under conventional conditions [12, 13].

Rearrangement reaction inherently involves the migration of atoms or chemical groups and the reorganization of the associated electrons [14, 15, 16]. Among the vast repertoire of rearrangements, the Cope rearrangement is a powerful synthetic principle that can be utilized to generate a C─C bond at an expense of a N─N bond [17]. The Cope rearrangement forms the basis of fundamental acid‐catalyzed transformations such as the Fischer idolization [18] and the benzidine rearrangement. The century‐long debates on the mechanism of benzidine rearrangement reaction was finally confirmed to be the [5,5] sigmatropic nature by the kinetic isotope experiments, albeit the possibility of different competing mechanisms [19, 20, 21, 22, 23, 24, 25]. This reaction has now been broadly applied in the preparation of biphenyls with or without the amino groups.

Here, we show that although hydrazobenzene itself cannot form molecular junctions with the Au electrodes and shows no conductance peak, in contrast, in the presence of acid, hydrazobenzene exhibits a well‐defined conductance peak at ∼1.3×10^−3^ G_0_. We propose that with the addition of acid, hydrazobenzenes undergo rearrangement reactions to form benzidines that form molecular junctions, supported by measurements of ex situ synthesized benzidines. We emphasize that although base is generally needed for obtaining benzidines from the rearrangement reaction mixture, in break‐junction experiments, Au‐benzidine‐Au junctions are seen with no addition of base, suggesting that the deprotonation reaction possibly occurs upon the junction formation. We further synthesize two series of hydrazobenzene derivatives: one characterized by the positional installation of paired methyl groups on the phenyl rings, and the other by systematic variation of the groups flanking the central ‐NH─NH‐ bond. We find that only when the methyl groups are introduced at the ortho positions relative to the ‐NH─NH‐ bond on the benzene, the rearrangement still proceeds; for meta‐ and para‐substituted derivatives, as well as derivatives containing biphenyl and naphthyl structures, rearrangement does not occur under identical conditions.

## 2 Results and Discussion

### 2.1 Synthesis of Hydrazobenzene Derivatives **2–6**

**SCHEME 1:**
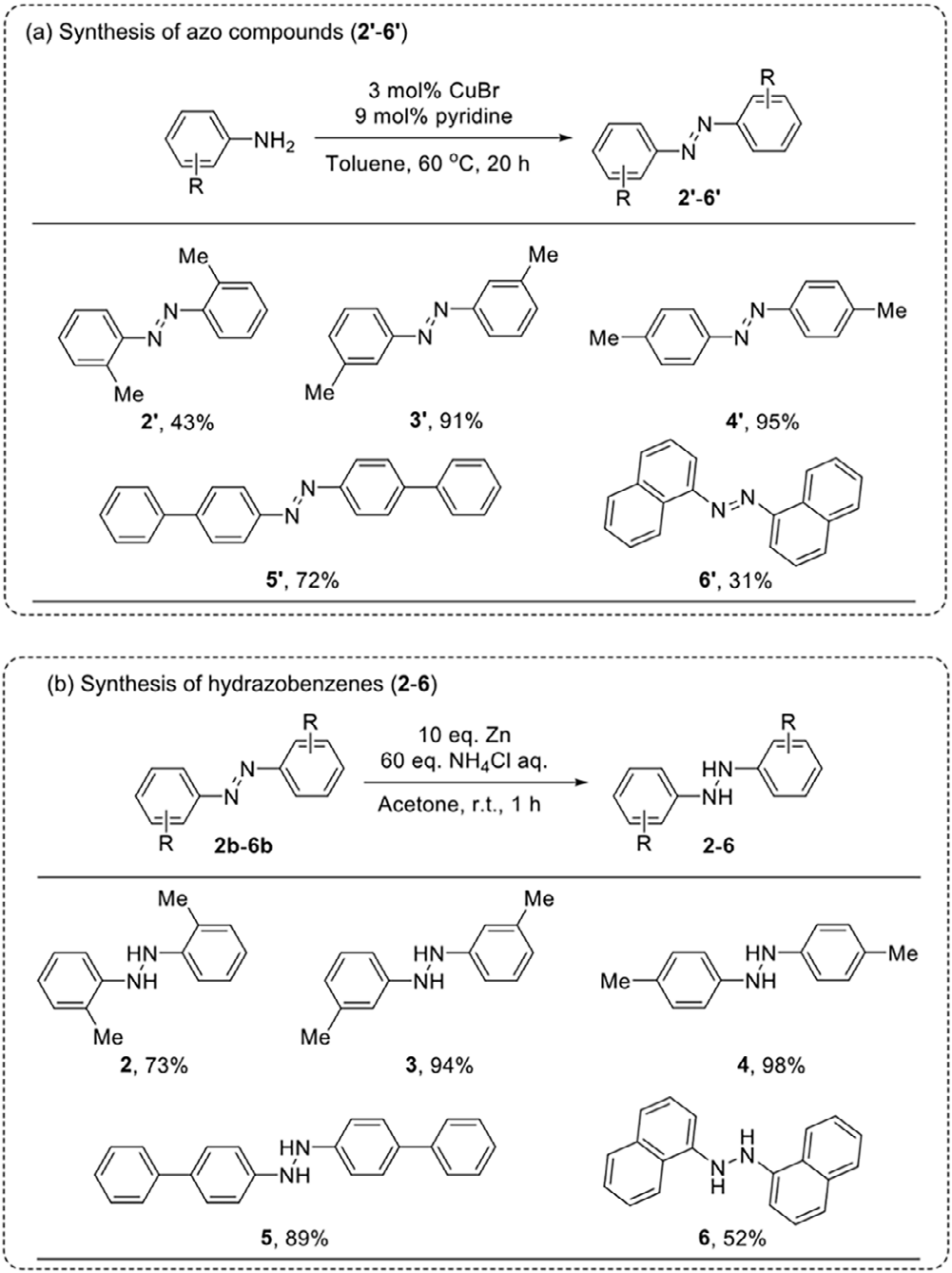
Synthetic route toward compounds **2**–**6**.

Using a well‐established method developed by the Jiao group [26], we successfully synthesized a series of azo compounds (**2’**‐**6’**) from various anilines, employing CuBr and pyridine as catalysts (Scheme 1a). Methyl groups were introduced at different positions on the aromatic ring, along with substitutions using biphenyl and naphthalene rings, resulting in yields ranging from 31% to 95%. Following this, the azo compounds were reduced to hydrazobenzene derivatives (**2**‐**6**) with moderate to good yields (52%–98%) using a Zn/NH_4_Cl reducing system (Scheme 1b). All products were purified using column chromatography on silica gel and characterized by NMR spectroscopy.

### 2.2 Acid‐Catalyzed Junction Formation for **1**

**FIGURE 1:**
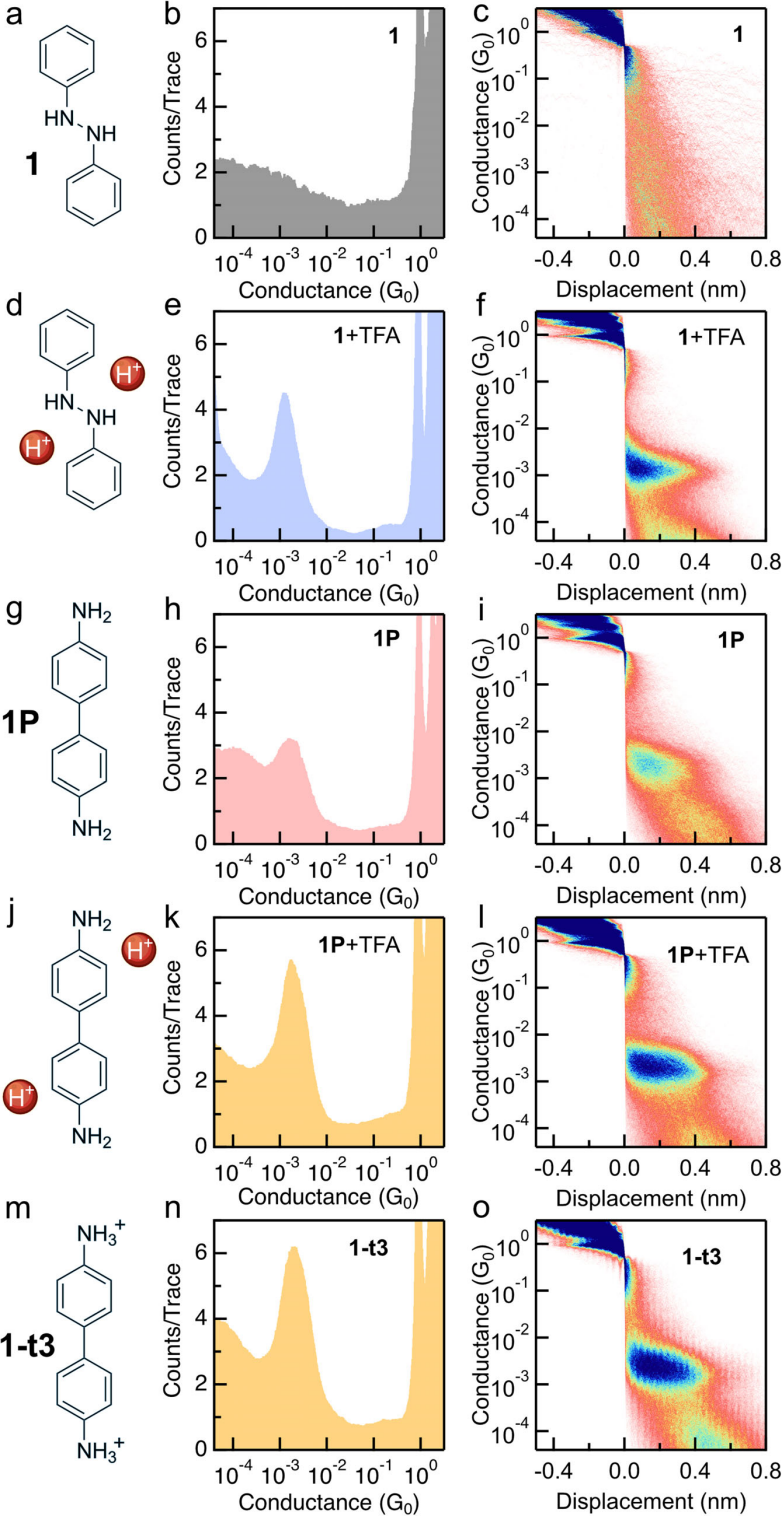
(a) Chemical structure for **1** (hydrazobenzene). (b) Logarithmically‐binned 1D and (c) 2D histograms of the conductance measurement of **1**. (d) Illustration of **1** with the addition of acid. (e) 1D and (f) 2D histograms of **1** measured in the presence of 1 equiv of trifluoroacetic acid (TFA). (g) Chemical structure for **1P** (benzidine). (h) 1D and (i) 2D histograms of the conductance measurement of **1P**. (j) Illustration of **1P** with the addition of acid. (k) 1D and (l) 2D histograms of **1P** measured in the presence of 50 equiv of TFA. (m) Chemical structures for the protonated ammonium salt of **1P**, which is named as **1‐t3**. (n) 1D and (o) 2D histograms of the conductance measurement of **1‐t3**.

We first study hydrazobenzene (**1**, chemical structure shown in Figure 1a). We perform measurements of **1** and, as anticipated, due to the absence of any anchoring groups, **1** does not exhibit any discernible conductance peaks in either one‐dimensional (1D) or two‐dimensional (2D) conductance histograms (Figure 1b,c), indicating that no benzidine (**1P**) was formed. Considering that acid serves as a catalyst for this reaction by facilitating the initial protonation of the nitrogens in **1** [19], we introduce an organic acid (Figure 1d), trifluoroacetic acid (TFA), in a 1:1 stoichiometric ratio relative to **1** in the nonpolar solvent 1,2,4‐trichlorobenzene (TCB) and perform the conductance experiment. Clear conductance plateau at ∼10^−3^ G_0_ in individual conductance traces are observed (Figure S1a), and a well‐defined conductance peak at 1.3×10^−3^ G_0_ is seen in the 1D histogram (Figure 1e). In the 2D histogram, we observe a ∼0.44 nm molecular junction elongation length (Figure 1f). We find that this conductance peak of **1** measured in the presence of TFA shows a similar conductance value and elongation length as those of ex situ synthesized **1P** (chemical structure in Figure 1g; 1.5 × 10^−3^ G_0_, 0.53 nm; Figure 1h,i). Based on these results, we hypothesize that in measurements of **1** with the addition of TFA, the conductance peak results from junctions of **1P** formed in situ.

Attempts have been made for confirming the reaction product **1P** using ex situ characterization methods, and the related data and detailed discussions are provided in Section S4. All of the methods discussed in the Supporting Information, including NMR, mass spectrometry, high‐performance liquid chromatography (HPLC), and Ultraviolet–visible spectroscopy, do not allow us to confirm if trace amount of **1P** is already generated in a nonpolar TCB solvent that contains TFA prior to any break‐junction experiments. Furthermore, although we cannot exclusively determine if Au plays any role in catalyzing this reaction, we find that an applied electric field might modestly promote this reaction (comparison of Figures S17 and S18). Next, we find that the rearrangement reaction of **1** occurs almost immediately in a break‐junction measurement (Figure S1b), even under a bias voltage as low as 5 mV (Figure S2a,b). We also find that when the TFA concentration is increased to 50 equiv, the reaction proceeds similarly to that of the experiment with the addition of 1 equiv of TFA (Figure S2c,d). In the absence of acid, even when polar and protic solvents such as water, ethanol, and methanol were used for STM‐BJ experiments, no single‐molecule junction conductance peaks were observed, as shown in Figure S3. This indicates that acid is a required condition for obtaining **1P** products, confirmed by HPLC analysis (Figures S14, S16, and S19).

In the benzidine rearrangement reaction, we emphasize that **1‐t3** (chemical structure is shown in Figure 1m) is formed in the presence of acid, and base is considered to be needed for obtaining the benzidine from **1‐t3** in a nonpolar solvent [24]. We suggest that protonated amines cannot form NH_2_→Au dative interactions with Au electrodes in forming junctions due to the absence of the lone pair of electrons on the nitrogen, as was observed by Chen et al. [27] that no molecular junctions are formed at pH = 1 for amine‐terminated alkanes. We note that here the measurements are performed in a nonpolar TCB solvent rather than in a polar H_2_O solvent, thus there might not be abundant supply of H^+^ and OH^−^ in our experiments, even with the addition of TFA. Our results of **1P** in the presence of 50 equiv of TFA and of ex situ synthesized **1‐t3** compound support this hypothesis, as both show a conductance and a junction elongation length the same as those of **1P** (Figure 1j–o). Given these observations, we propose that either the presence of the Au atoms, or the electric field, or both, possibly facilitates the deprotonation of **1‐t3** in TCB into forming junctions linked by NH_2_→Au dative interactions.

**FIGURE 2:**
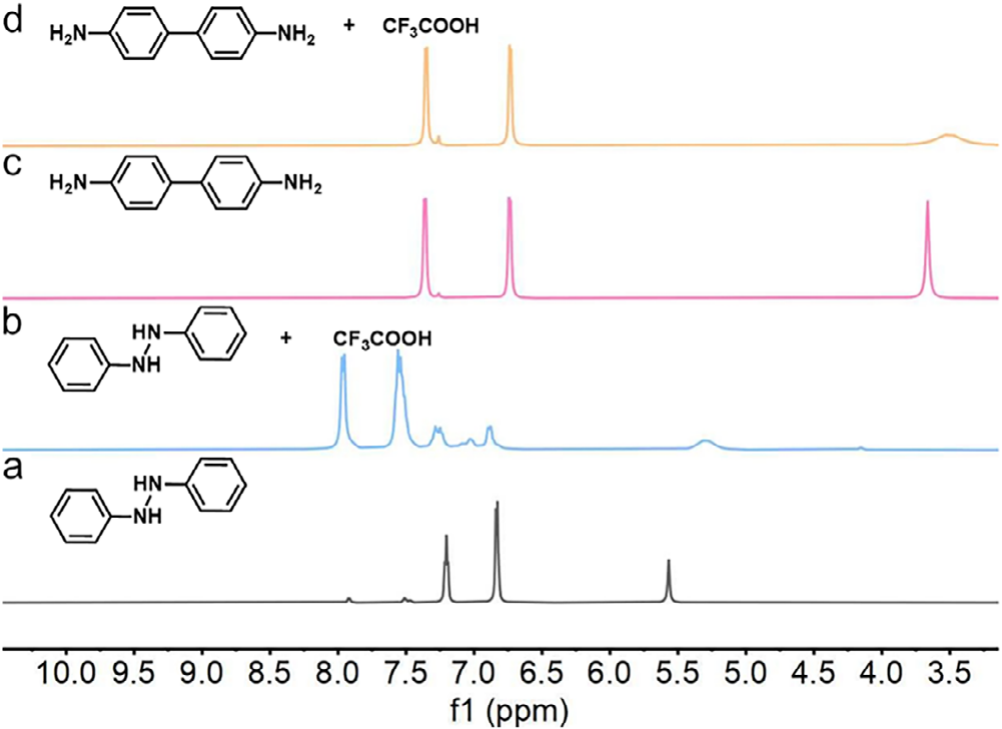
^1^H NMR spectra of 15 mg/ml compound **1** in solvent CDCl_3_ (a) before and (b) after TFA addition. ^1^H NMR spectra of 15 mg/ml compound **1P** in CDCl_3_ (c) before and (d) after TFA addition.

While the acid‐catalyzed rearrangement of hydrazobenzene to benzidine is well‐established, it typically requires polar protic solvents (e.g., aqueous acids, acetic acid, or alcohols). In contrast, we confirmed that neutral solvents like CDCl_3_ or benzene are unsuitable for this transformation. As shown in Figure 2, the addition of TFA to hydrazobenzene in the absence of a polar protic solvent and without the break‐junction measurements fails to produce a detectable amount of benzidine **1P** in NMR. Specifically, in the ^1^H NMR spectra, in the presence of TFA, the aromatic protons of hydrazobenzene shifted to signals at 7.5 and 8.0 ppm, which differ significantly from those of compound **1** (6.7 and 7.3 ppm). This result provides clear evidence that TFA alone cannot efficiently catalyze the rearrangement of **1** to **1P** on a large scale.

### 2.3 Acid‐Catalyzed Junction Formation for **2**

**FIGURE 3:**
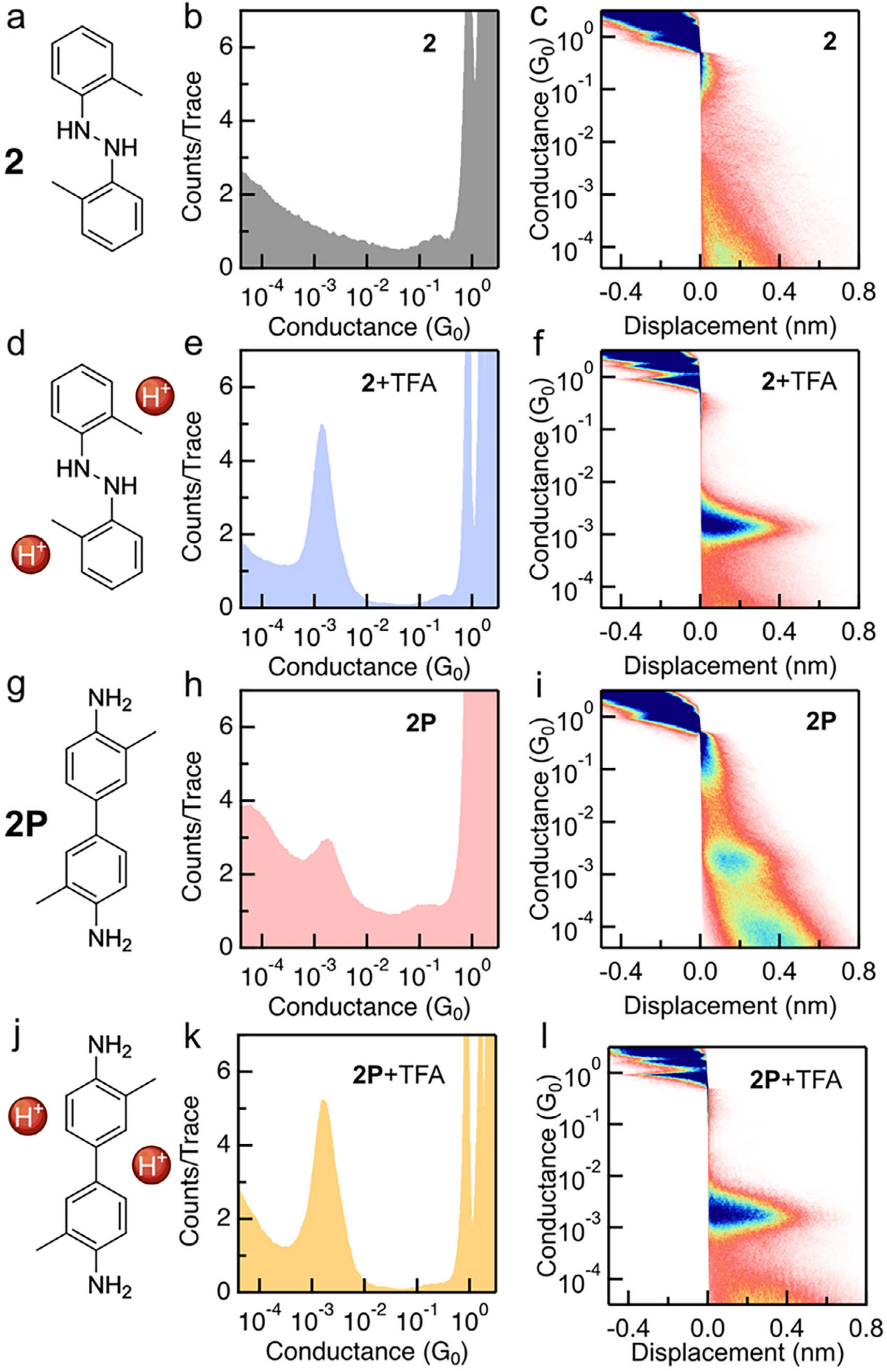
(a) Chemical structure for **2**. (b) Logarithmically‐binned 1D and (c) 2D histograms of the conductance measurement of **2**. (d) Illustration of **2** with the addition of acid. (e) 1D and (f) 2D histograms of **2** measured in the presence of 1 equiv of trifluoroacetic acid (TFA). (g) Chemical structure for **2P**. (h) 1D and (i) 2D histograms of the conductance measurement of **2P**. (j) Illustration of **2P** with the addition of acid. (k) 1D and (l) 2D histograms of **2P** measured in the presence of 50 equiv of TFA.

**FIGURE 4:**
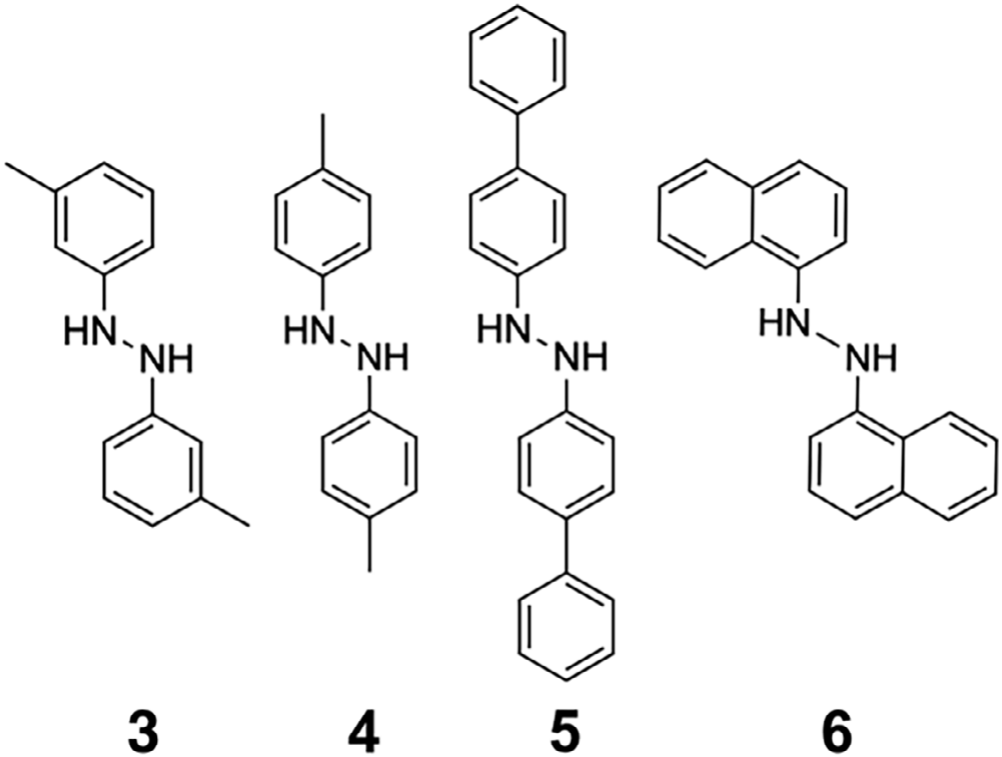
Chemical structures of compound **3**–**6**, which do not exhibit a conductance peak in STM‐BJ experiments when acid was added.

Next, we introduce two methyl substituents on the two phenyl rings of **1** and obtain three derivatives: ortho‐substituted (**2**), meta‐substituted (**3**), and para‐substituted (**4**) analogues, the molecular structures of which are depicted in Figure 3a (**2**) and Figure 4 (**3** and **4**). Consistent with the behavior of **1**, compounds **2**–**4** exhibit no molecular conductance peaks when measured in the absence of acid, as shown in Figures 3b,c, and S4. With the addition of one equiv of TFA in the measurement of **2**, we see molecular plateaus (example traces shown in Figure S5a) and observe a well‐defined conductance peak at 1.4×10^−3^ G_0_ with elongation length of 0.39 nm (Figure 3e,f). Since **2** itself cannot form junctions due to the lack of any linker groups that bind to gold, these results suggest that, under acidic conditions, the rearrangement of **2** occurs and the product **2P** is formed (structure shown in Figure 3g). Indeed, this conductance signature agrees with that of ex situ synthesized **2P**, which shows a 1.5×10^−3^ G_0_ conductance with 0.46 nm junction elongation length (Figure 3h,i), as well as that of **2P** measured in the presence of 50 equiv of TFA (Figure 3k,l). We notice an intriguing phenomenon in both measurements of **1** and **2** that the conductance peak of the acid‐catalyzed reaction products is narrower and more well‐defined in comparison to that of the measurements of **1P** and **2P**, respectively. We find that the reaction for **2P** occurs as soon as we start the break‐junction experiments, as shown in Figure S5b. We note that in the measurements of **1P** and **2P**, we both observe a second low conductance peak at ∼9.7 × 10^−5^ G_0_ (ended at ∼0.81 nm) and ∼4.7×10^−5^ G_0_ (ended at ∼0.70 nm) respectively, which may be attributed to the intermolecular π–π stacked junctions [28, 29, 30].

### 2.4 No Junction Formation Observed for **3–6**

For experiments of the meta‐ and para‐substituted analogues **3** and **4** in the presence of TFA, no discernible conductance peaks are observed in the 1D or 2D histograms, as shown in Figures S6 and S7. The proposed rearrangement product for **3** is labeled as **3P** (chemical structure is given in Figure S8a), and ex situ synthesized **3P** shows a clear conductance peak at 4.7×10^−5^ G_0_ (Figures S8b,c). The lack of conductance features in the measurements of **3** and **4** suggests that, under identical experimental conditions with the applied electric field and supplied acid, **3** and **4** do not undergo rearrangement reactions or the reactions do not proceed to forming the amine‐terminated compounds. We also note that acid may have other effects in addition to the catalyzation of the rearrangement reaction, such as protonation of the secondary amines present in **1**–**3**, or affect the charge transport as solvent molecules surrounding the single‐molecule junction. These results of **3**/**4** + TFA (as well as **5**/**6** + TFA that will be discussed below) that do not show any conductance peak further rule out the possibility of other impacts of acid in enabling the junction formation in measurements of **1**/**2** + TFA.

We further synthesize hydrazobenzene derivatives **5** and **6**, which contain biphenyls and naphthyls in the backbone, respectively (structures are given in Figure 4). Given the molecular length of the expected products of the rearrangement reaction of **5** and **6**, we expect that if they are formed, they show a low molecular conductance. We hypothesize that the concerted sigmatropic reaction of **5** could theoretically proceed along the biphenyl structure, with the expected product quaterphenyl‐4,4'''‐diamine (**5P**, chemical structure shown in Figure S9a). The ex situ synthesized **5P** shows a single‐molecule junction conductance of 7.7×10^−5^ G_0_ (Figure S9b,c). No characteristic conductance peak is observed in the STM‐BJ experiments of **5** (Figure S10), suggesting that in STM‐BJ experiments with protons present, rearrangement of **5** either does not occur or does not proceed to completion. This lack of reactivity may be attributed to the steric constraints imposed by the bulky biphenyl moieties, which likely hinder the conformational reorganization necessary for the formation of the bridging C─C bond at the para‐position. We again do not see a conductance peak for **6** under an applied electric field with the addition of protons (Figure S11), indicating that the expected amine‐linked molecular junctions are not formed. Given our observation, σ‐bond migration for forming the C─C bond is not successfully accomplished in a larger π‐conjugated structure of naphthalene.

### 2.5 Mechanism for Junction Formation of **1–3**

**FIGURE 5:**
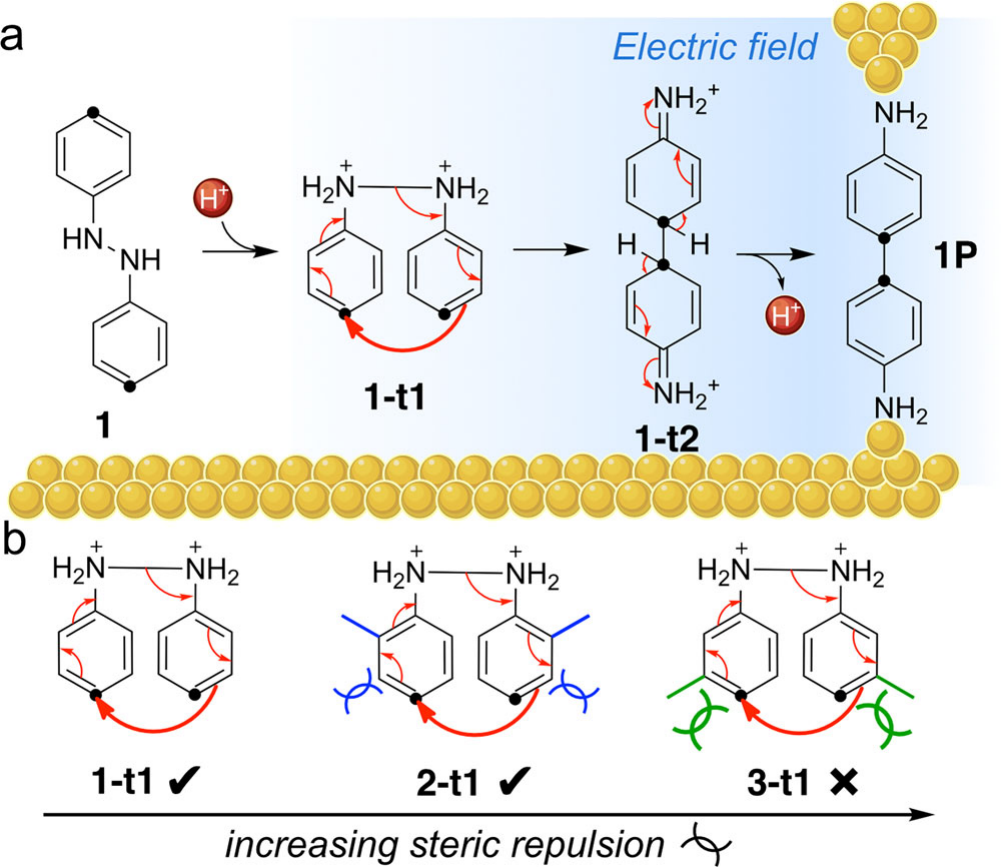
(a) The proposed **1**→**1P** rearrangement reaction mechanism in STM‐BJ experiments. (b) Comparison of the transition states for **1**, **2**, and **3**, labeled as **1‐t1**, **2‐t1**, and **3‐t1**, respectively.

To understand the observed different reactivities among **1**–**4**, we refer to the mechanism, which suggests that the rearrangement occurs via cationic intermediates, as illustrated in Figure 5a [25, 31]. In the presence of TFA in the solution, both N atoms in **1** undergo initial protonation, forming ‐NH_2_^+^‐ groups, as shown as **1‐t1**. This protonation facilitates the cleavage of the N─N σ‐bond, followed by the σ‐bond migration to one of the phenyl rings to form a C═N double bond. Concurrently, a σ‐bond within the same phenyl ring migrates to the adjacent position, ultimately leading to the formation of a new C─C σ‐bond between the two phenyl rings. The resulting intermediate contains an additional hydrogen atom on the bridging carbons, and this C─H σ‐bond subsequently undergoes intramolecular migration along the phenyl ring. It should be noted that the structure denoted as **1‐t3** before is one of the resonance forms of the intermediate **1‐t2** here. The final deprotonation step to form **1P** usually requires the addition of base. The entire process to obtain **1P** has been demonstrated to occur via a concerted [5,5]‐sigmatropic mechanism [19, 32]. Previous studies have revealed that this reaction is accompanied by the formation of several byproducts (full reaction equation is given in Figure S12) [33]. Among the four byproducts **7**–**10** (chemical structures in Figure S12), we carried out conductance experiments of **7** (synthesis and characterization of **7** are provided in Section S1, and NMR of **7** is given in Section S5) and **8** (Macklin, ≥ 99%), and observe no conductance peaks (Figure S13). For **9** and **10**, only one chemical linker group is present thus both compounds cannot form stable molecular junctions with Au electrodes. In addition, apart from **7** which was reported to be of considerable amount (15%–30%), the amount of **8**, **9**, and **10** is expected to be significantly lower than that of **1P** [31, 33]. Therefore, this study focuses primarily on the formation and characterization of the main reaction product **1P**.

We next compare the three **t1** states for **1–3** (Figure 5b). For the phenyl rings in the unsubstituted **1**, we consider that no steric hindrance is present. In ortho‐substituted **2**, substituents not only appear to facilitate the central N─N bond cleavage but also introduce some level of steric hindrance to the formation of the bridging C─C bond [24]. Overall, we still see a successful rearrangement reaction for **2**. In contrast, the substituents on the meta positions in **3** are closer to the reacting carbons, exhibiting a stronger steric blockage effect than those on **2** (illustrated in Figure 5b), which ultimately impede the connection between the two carbons and inhibit the formation of **3P**.

## 3 Conclusion

In summary, we have demonstrated that acid catalyzes the formation of robust single‐molecule junctions in STM‐BJ experiments of hydrazobenzenes **1** and **2**. The single‐molecule device formation is directly monitored via the emergence of a well‐defined conductance peak, which is not occurring in the absence of acid. Importantly, we find that the tendency of forming the NH_2_→Au dative interactions between the amines and the Au electrodes under a bias voltage possibly facilitates the deprotonation of the transition states into producing the amine‐terminated benzidine products. NMR data indicate that in the presence of TFA in a nonpolar solvent, the rearrangement reaction of **1** does not occur on a large scale. Our findings of **3–6** reveal that the substitution groups on the meta and para positions of the phenyl rings of hydrazobenzene and the extended conjugated structures installed in hydrazobenzene will inhibit the rearrangement reaction. This study provides critical mechanistic insights into the reactivity of hydrazobenzenes in rearrangement reactions and establishes a strategy for single‐molecule junction formation promoted by the use of acid.

## Conflicts of Interest

The authors declare no conflicts of interest.

## Supporting information



The authors have cited additional references within the Supporting Information [33, 34, 35].

## Data Availability

The data that support the findings of this study are available in the Supporting Information of this article.
