# Supplementary material for: Acid‐Catalyzed Rearrangement Reaction for Single‐Molecule Junction Formation
Source: Chemistry. 2026 Jun 6;32(28):e71233. doi: 10.1002/chem.71233 (PMC13411208; doi:10.1002/chem.71233)
Supplement: Supplementary file 1 — The authors have cited additional references within the Supporting Information [33, 34, 35]. [file CHEM-32-e71233-s001.docx]

**Supporting information**

Acid-catalyzed rearrangement reaction for single-molecule junction formation

**Yihao Zhang^1#^, Yunlong Li^2#^, Zhenpin Lu^2^*,** Haixing Li^1^*

^1^Department of Physics, City University of Hong Kong, Kowloon 999077, Hong Kong SAR, China

^2^Department of Chemistry, City University of Hong Kong, Kowloon 999077, Hong Kong SAR, China

**^#^These authors contributed equally.**

**Table of Contents**

**Section 1.** Synthetic procedures and characterization of compounds

**Section 2.** Scanning tunneling microscope-based break junction experiment details

**Section 3.** Additional figures

**Section 4.** Ex situ analysis of the reaction products

**Section 5.** NMR spectra

**Section 6.** References

**Section 1. Synthetic procedures and characterization of compounds**

General procedure for the synthesis of azo compounds 2’-6’

**A typical procedure for the synthesis of (*E*)-1,2-di-*o*-tolyldiazene (2’)**: A mixture of *o*-toluidine (161 mg, 1.5 mmol), CuBr (6.5 mg, 0.045 mmol), and pyridine (11 mg, 0.135 mmol) in 6 mL of toluene was stirred at 60 ^o^C for 20 h. The reaction mixture was poured into water (6 ml) and extracted with DCM (12 mL), the organic layer was separated and dried over anhydrous sodium sulfate, all the volatiles were removed under vacuum condition. The resulting mixture was purified by column chromatography on silica gel (eluent: hexane/dichloromethane = 5:1, v/v), yielding compound **2’** as a yellow solid (68 mg, 43%).

**^1^H NMR** (600 MHz, CDCl_3_) *δ* 7.64 – 7.60 (m, 2H), 7.34 – 7.29 (m, 4H), 7.25 – 7.22 (m, 2H), 2.73 (s, 6H); **^13^C NMR** (151 MHz, CDCl_3_) *δ* 151.20, 138.11, 131.37, 130.79, 126.46, 115.96, 17.73.

**(*E*)-1,2-di-*m*-tolyldiazene (3’)**: 143.5 mg, 91% yield, yellow solid. **^1^H NMR** (600 MHz, CDCl_3_) *δ* 7.71 – 7.64 (m, 4H), 7.30 – 7.26 (m, 2H), 7.17 – 7.12 (m, 2H), 2.33 (s, 6H); **^13^C NMR** (151 MHz, CDCl_3_) *δ* 152.81, 138.86, 131.70, 128.88, 123.01, 120.51, 21.36.

**(*E*)-1,2-di-*p*-tolyldiazene (4’)**: 149.8 mg, 95% yield, yellow solid. **^1^H NMR** (600 MHz, CDCl_3_) *δ* 7.86 (d, *J* = 8.1 Hz, 4H), 7.34 (d, *J* = 8.0 Hz, 4H), 2.46 (s, 6H); **^13^C NMR** (151 MHz, CDCl_3_) *δ* 150.95, 141.28, 129.81, 122.85, 21.58.

**(*E*)-1,2-di([1,1'-biphenyl]-4-yl)diazene (5’)**: 180.6 mg, 72% yield, yellow solid. **^1^H NMR** (400 MHz, CDCl_3_) *δ* 8.03 (d, *J* = 8.5 Hz, 4H), 7.79 – 7.74 (m, 4H), 7.71 – 7.66 (m, 4H), 7.52 – 7.46 (m, 4H), 7.43 – 7.37 (m, 2H); **^13^C NMR** (151 MHz, CDCl_3_) *δ* 152.06, 143.89, 140.39, 129.07, 128.06, 127.96, 127.37, 123.56.

**(E)-1,2-di(naphthalen-1-yl)diazene (6’)**: 65.6 mg, 31% yield, yellow solid. **^1^H NMR** (600 MHz, CDCl_3_) *δ* 9.06 (d, *J* = 8.4 Hz, 2H), 8.03 (d, J = 7.4 Hz, 2H), 8.00 (d, *J* = 7.5 Hz, 2H), 7.96 (d, *J* = 8.2 Hz, 2H), 7.71 – 7.67 (m, 2H), 7.65 – 7.60 (m, 4H); **^13^C NMR** (151 MHz, CDCl_3_) *δ* 148.41, 134.53, 131.66, 131.56, 128.11, 127.09, 126.64, 125.81, 123.75, 112.43.

General procedure for the synthesis of hydrazobenzene derivatives 2-6

**A typical procedure for the synthesis of 1,2-di-*o*-tolylhydrazine (2)**: A mixture of **(*E*)-1,2-di-*o*-tolyldiazene** (67.5 mg, 0.32 mmol), Zn (209.2 mg, 3.2 mmol), and NH_4_Cl (1027 mg, 19.2 mmol) in 14 mL of acetone/H_2_O (2.5:1, v/v) was stirred at 25 ^o^C for 1 h. The reaction mixture was then poured into water (20 mL) and extracted with DCM (40 mL). The organic layer was separated and dried over anhydrous sodium sulfate, and all volatiles were evaporated under reduced pressure. The resulting mixture was purified by column chromatography on silica gel (eluent: hexane/dichloromethane = 2:1, v/v), yielding compound **2** as a yellow solid (49.6 mg, 73%).

**^1^H NMR** (400 MHz, CDCl_3_) *δ* 7.29 – 7.21 (m, 4H), 7.08 – 7.01 (m, 2H), 6.98 – 6.90 (m, 2H), 5.63 (s, 2H), 2.39 (s, 6H); **^13^C NMR** (101 MHz, CDCl_3_) *δ* 146.33, 130.45, 127.32, 121.23, 119.43, 111.02, 17.25.

**1,2-Di-*m*-tolylhydrazine (3)**: 63.9 mg, 94% yield, yellow solid. **^1^H NMR** (600 MHz, CDCl_3_) *δ* 7.41 – 7.24 (m, 2H), 7.01 – 6.73 (m, 6H), 5.64 (s, 2H), 2.51 (s, 6H); **^13^C NMR** (151 MHz, CDCl_3_) *δ* 149.14, 139.22, 129.24, 120.72, 113.00, 109.52, 21.64.

**1,2-Di-*p*-tolylhydrazine (4)**: 66.6 mg, 98% yield, yellow solid. **^1^H NMR** (600 MHz, CDCl_3_) *δ* 7.41 (s, 4H), 7.12 (s, 4H), 5.80 (s, 2H), 2.69 – 2.60 (m, 6H); **^13^C NMR** (151 MHz, CDCl_3_) *δ* 146.83, 129.86, 128.99, 112.54, 20.56.

**1,2-Di([1,1'-biphenyl]-4-yl)hydrazine (5)**: 95.8 mg, 89% yield, yellow solid. **^1^H NMR** (400 MHz, CDCl_3_) *δ* 7.59 (d, *J* = 6.6 Hz, 4H), 7.53 (d, *J* = 7.3 Hz, 4H), 7.44 (s, 4H), 7.36 – 7.30 (m, 2H), 6.99 (d, *J* = 7.3 Hz, 4H), 5.77 (s, 2H); **^13^C NMR** (101 MHz, CDCl_3_) *δ* 148.30, 141.09, 133.15, 128.83, 128.24, 126.63, 126.58, 112.81.

**1,2-Di(naphthalen-1-yl)hydrazine (6)**: 47.3 mg, 52% yield, yellow solid. **^1^H NMR** (600 MHz, CDCl_3_) *δ* 7.96 – 7.93 (m, 2H), 7.90 – 7.86 (m, 2H), 7.55 – 7.51 (m, 4H), 7.39 (d, *J* = 8.1 Hz, 2H), 7.30 – 7.27 (m, 2H), 7.03 (d, *J* = 7.6 Hz, 2H), 6.39 (s, 2H); **^13^C NMR** (101 MHz, CDCl_3_) *δ* 142.86, 134.44, 129.00, 126.65, 126.09, 125.41, 122.63, 120.24, 119.82, 106.39.

General procedure for the synthesis of 4’-nitro-[1,1’-biphenyl]-2-amine (7’)^1^

**A typical procedure for the synthesis of 4’-nitro-[1,1’-biphenyl]-2-amine (7’)**: A mixture of **[1,1’-biphenyl]-2-amine** (457 mg, 2.7 mmol), H_2_SO_4_ (4.3 mL), and HNO_3_ (0.11 mL) was stirred at -5 ^o^C for 2 h. The reaction mixture was poured into ice-water mixture (20 ml) and saturated sodium hydroxide solution (10 mL), filtered the suspension, washed with water, and then recrystallized with ethanol, yielding **4’-nitro-[1,1’-biphenyl]-2-amine (7’)** as a yellow solid (401 mg, 69%).

**^1^H NMR** (300 MHz, CDCl_3_) *δ* 8.30 (d, *J* = 8.3 Hz, 2H), 7.67 (d, *J* = 8.3 Hz, 2H), 7.26 – 7.20 (m, 1H), 7.13 (d, *J* = 7.5 Hz, 1H), 6.90 – 6.85 (m, 1H), 6.80 (d, *J* = 8.0 Hz, 1H), 3.81 (s, 2H); **^13^C NMR** (75 MHz, CDCl_3_) *δ* 146.97, 146.74, 143.40, 130.40, 130.05, 129.96, 125.20, 124.23, 119.23, 116.34.

General procedure for the synthesis of [1,1’-biphenyl]-2,4’-diamine (7)^2^

**A typical procedure for the synthesis of [1,1’-biphenyl]-2,4’-diamine (7)**: A mixture of **4’-nitro-[1,1’-biphenyl]-2-amine** (64 mg, 0.3 mmol), SnCl_2_ (114 mg, 0.6 mmol), and HOAc (2.4 mL) was stirred at 110 ^o^C for 5 h. The reaction mixture was poured into saturated sodium hydroxide solution (8 mL), the reaction mixture was extracted with DCM (20 mL), the organic layer was separated and dried over anhydrous sodium sulfate, all volatiles were evaporated under reduced pressure. The resulting mixture was purified by column chromatography on silica gel (eluent: dichloromethane), yielding **[1,1’-biphenyl]-2,4’-diamine (7)** as a grey liquid (30.4 mg, 55%).

**^1^H NMR** (300 MHz, CDCl_3_) *δ* 7.29 – 7.24 (m, 2H), 7.16 – 7.11 (m, 2H), 6.85 – 6.79 (m, 1H), 6.79 – 6.72 (m, 3H), 3.63 (s, 4H); **^13^C NMR** (75 MHz, CDCl_3_) *δ* 145.60, 143.79, 130.51, 130.09, 129.58, 127.96, 127.92, 118.67, 115.54, 115.41.

**Section 2. Scanning tunneling microscope-based break junction experiment details**

Single-molecule conductance measurements were performed using a custom-built scanning tunneling microscope-based break junction (STM-BJ) technique^3, 4^ that has been described in detail in the supporting information of a previous work.^5^ We deposited ~100 nm thick gold (99.999%, Shijiazhuang Huake Metal Materials Technology Co., Ltd., China) thin film on a steel surface by the use of a thermal evaporation system (Beijing Technol Science Co Ltd, China) for use as the Au substrate. This substrate was treated with UV-Ozone for 20 min prior to each experiment. A freshly cut Au wire (Ø = 0.25 mm, 99.999%, ZhongNuo Advanced Material (Beijing) Technology Co., Ltd., China) was used as the STM tip.

Solutions of 0.1–1 mM target molecule in the solvent TCB were dropped onto the substrate for molecular conductance measurements. 1,2,4-trichlorobenzene (TCB) was purchased from Alfa Aesar (≥ 99%) or Aladdin (anhydrous, ≥ 99%). Trifluoroacetic acid (TFA) was purchased from Energy (≥ 99%). Hydrazobenzene (**1**) and benzidine (**1P**) were purchased from Aladdin (≥ 95%). 4,4'-Diamino-3,3'-dimethylbiphenyl (**2P**) and m-tolidine (**3P**) were purchased from Energy (98%). [1,1':4',1'':4'',1'''-Quaterphenyl]-4,4'''-diamine (**5P**) was purchased from Leyan (98%). The tip was displaced at a speed of 18 nm/s (15 nm/s for the 5 mV experiment in Figure S2) and the current and voltage data were acquired at the 40 kHz acquisition rate for all measurements.

**Section 3. Additional figures**


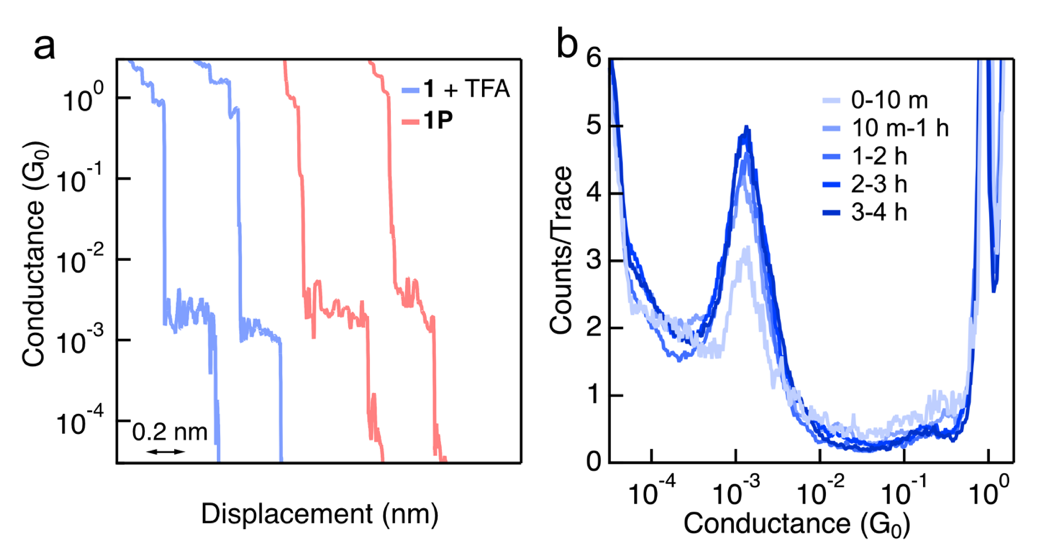


**Figure S1.** (a) Example traces for **1** measured with addition of 1 equiv of TFA (blue) and for **1P** (pink). (b) 1D histograms for **1** measured with addition of 1 equiv of TFA at 100 mV in the 0-10 m, 10 m-1 h, 1-2 h, 2-3 h, and 3-4 h time periods.


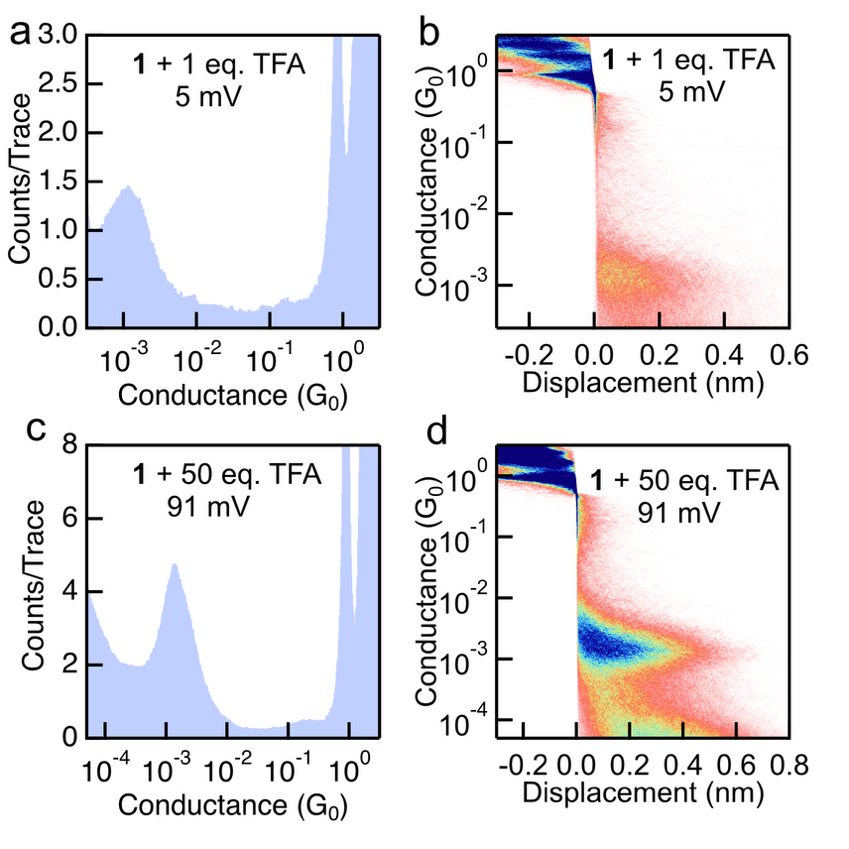


**Figure S2.** (a-b) (a) 1D and (b) 2D histograms of **1** measured with addition of 1 equiv of TFA at 5 mV. (c-d) (c) 1D and (d) 2D histograms of **1** measured with addition of 50 equiv of TFA at 91 mV.


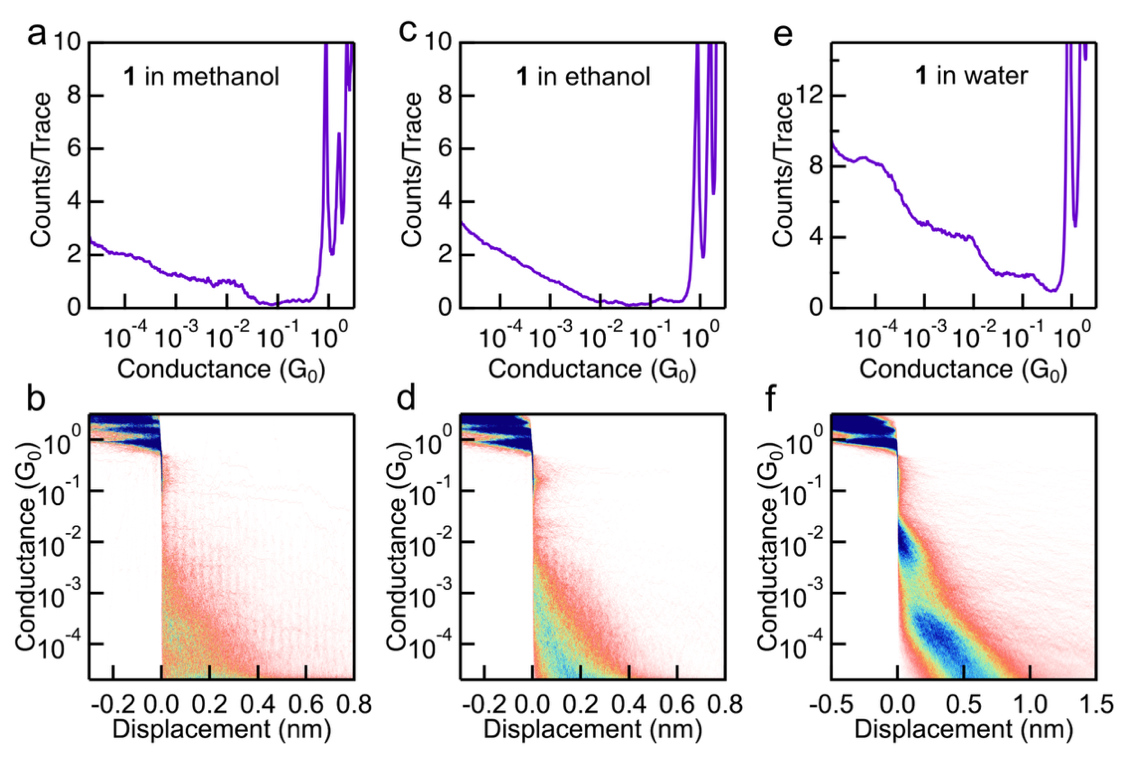


**Figure S3.** (a, c, e) 1D and (b, d, f) 2D histograms of **1** measured in (a, b) methanol, (c, d) ethanol, and (e, f) water without the addition of TFA.


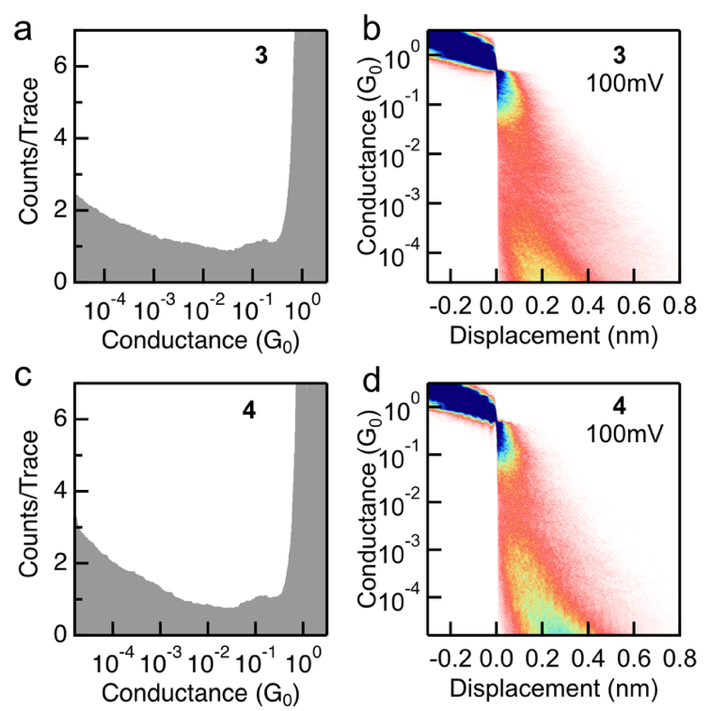


**Figure S4.** (a) 1D and (b) 2D histograms for **3** measured at 100 mV. (c) 1D and (d) 2D histograms for **4** measured at 100 mV.


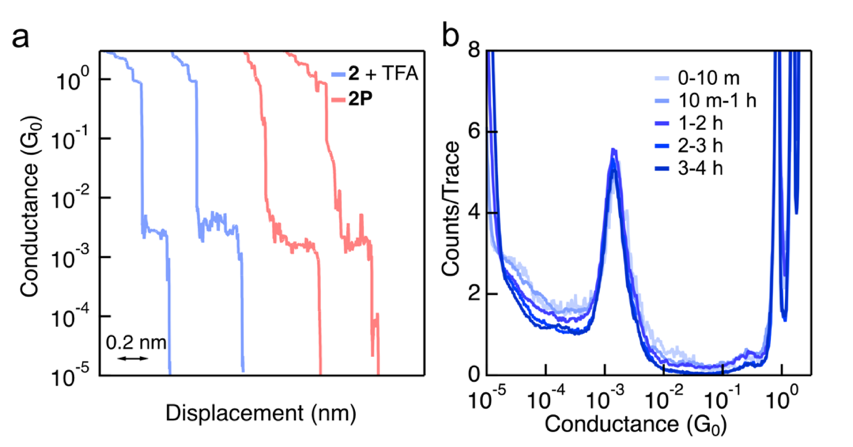


**Figure S5.** (a) Example traces for **2** measured with the addition of 1 equiv of TFA (blue) and for **2P** (pink). (b) 1D histograms for **2** measured with the addition of 1 equiv of TFA under 100 mV bias voltage in the 0-10 min, 10 min-1 h, 1-2 h, 2-3 h, and 3-4 h time periods.


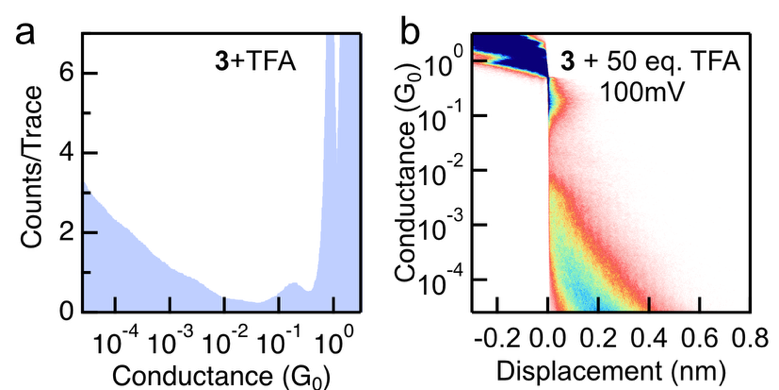


**Figure S6.** (a) 1D and (b) 2D histograms for **3** measured with the addition of 50 equiv of TFA at 100 mV.


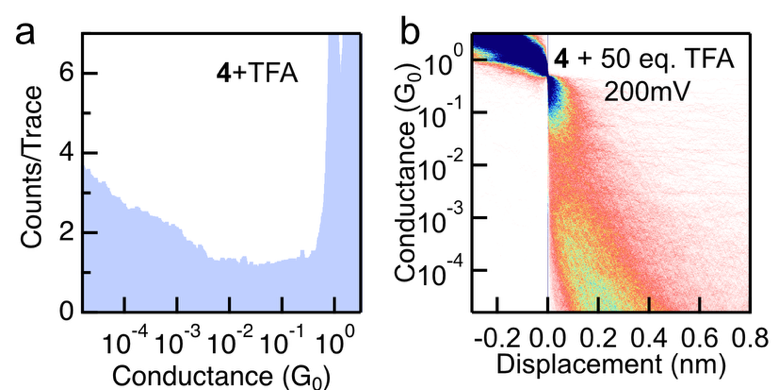


**Figure S7.** (a) 1D and (b) 2D histograms for **4** measured with the addition of 50 equiv of TFA at 200 mV.


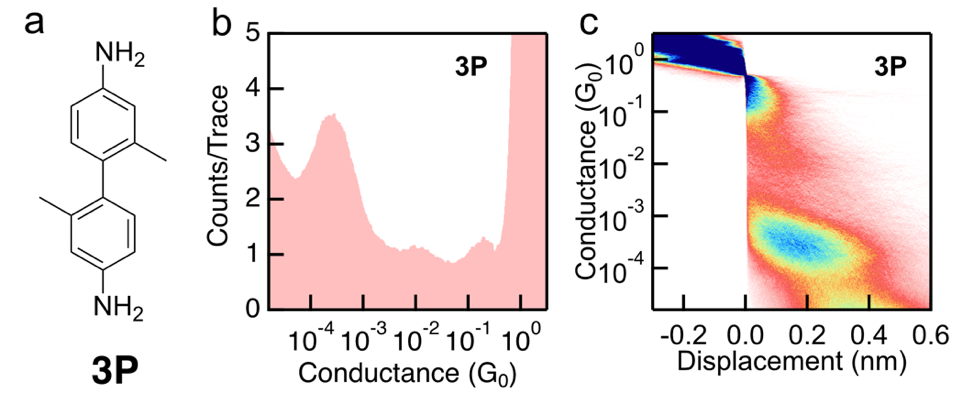


**Figure S8.** (a) Chemical structure for the proposed product **3P** for the rearrangement reaction of **3**. (b) 1D and (c) 2D histograms of **3P** measured at 91 mV.


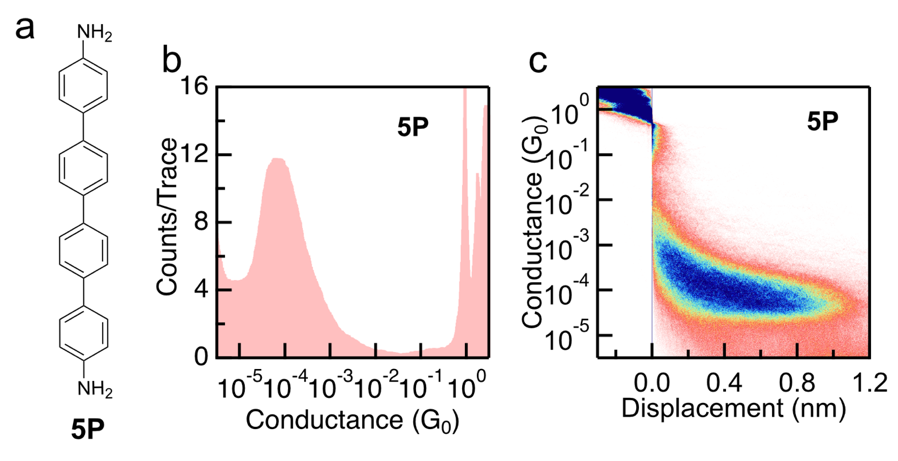


**Figure S9.** (a) Chemical structure for the expected product **5P** for the rearrangement reaction of **5**. (b) 1D and (c) 2D histograms of **5P** measured at 800 mV.


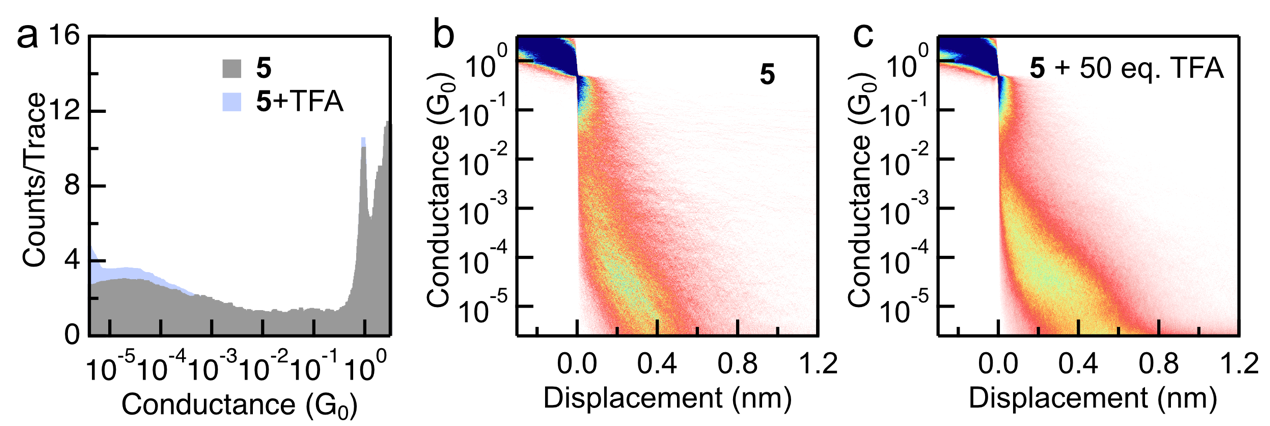


**Figure S10.** (a) 1D histograms of **5** and **5** with the addition of 50 equiv of TFA measured under 1 V. (b-c) 2D histograms of (b) **5** and (c) **5** with the addition of 50 equiv of TFA.


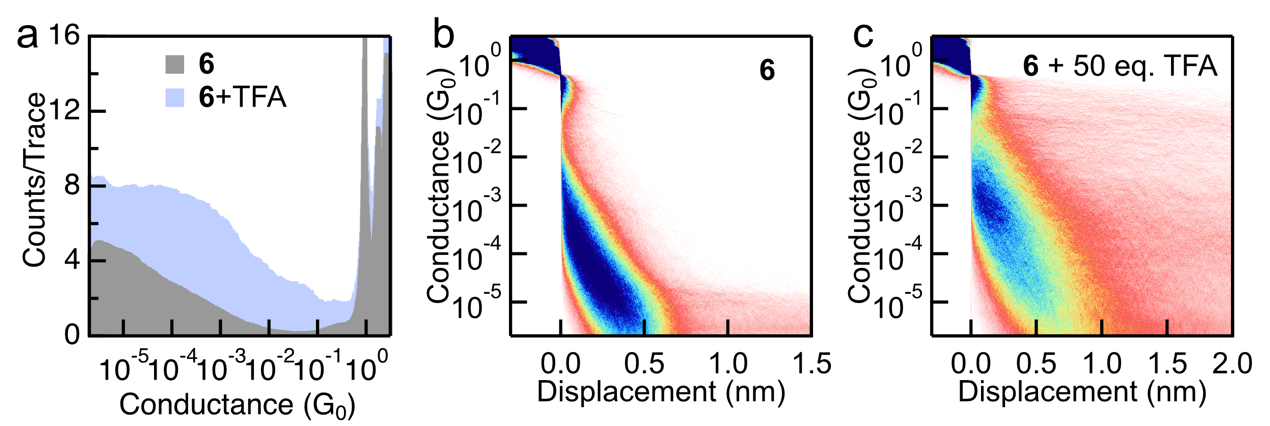


**Figure S11.** (a) 1D histograms of **6** and **6** with the addition of 50 equiv of TFA measured under 1 V. (b-c) 2D histograms of (b) **6** and (c) **6** with the addition of 50 equiv of TFA.

**
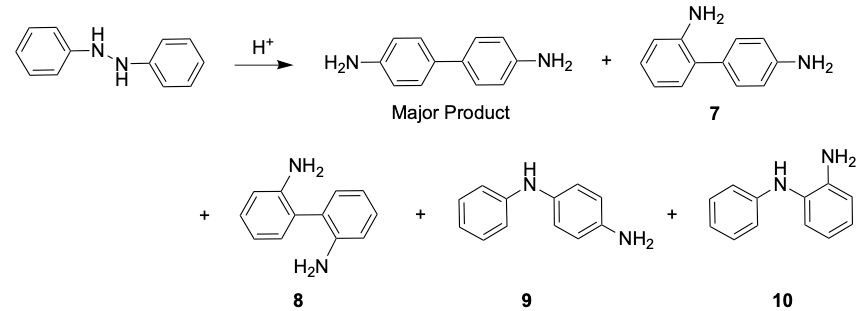
**

**Figure S12.** Structures of the four additional byproducts^6^ **7**, **8**, **9**, and **10** in rearrangement reaction of **1**.


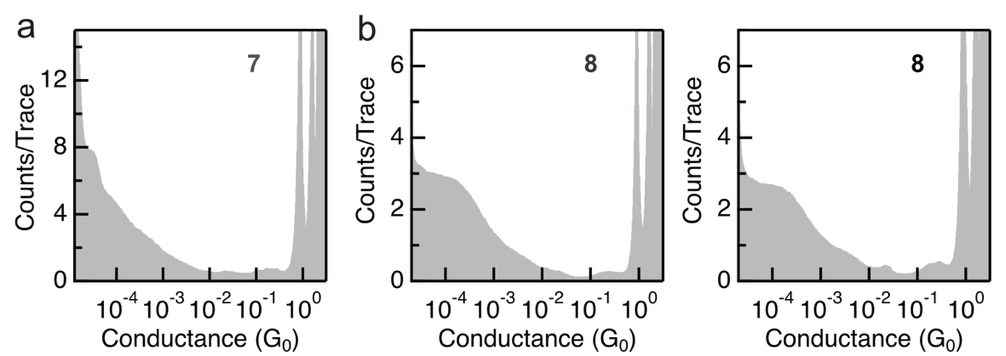


**Figure S13.** 1D histograms of (a) the conductance experiment of byproduct **7** measured at 100 mV, and (b) two repeated conductance experiments of the byproduct **8** measured at 100 mV.

**Section 4. Ex situ analysis of the reaction products**

We have considered the following ex situ characterization methods for conforming the rearrangement reaction products.

Method 1: NMR

For NMR, the detection limit is ~2 mg/ml. In our STM-BJ experiments, the compound (reactant) has a concentration of ~ 1 mM (0.184 mg/ml), below the detection limit of NMR method.

Method 2: Mass spectrometry

For the reactants and the products:

The chemical formula for both chemicals is C_12_H_12_N_2_. The mass for both compounds is 184.24. Thus, we cannot distinguish the two compounds by mass spectrometry.

Method 3: High-performance liquid chromatography (HPLC)

Chromatographic analysis was performed on Agilent 1260 HPLC system using an Agilent 5 HC-C18 column (250 × 4.6 mm). The separation was achieved via isocratic elution with a mobile phase consisting of 70% acetonitrile and 30% water (v/v). The flow rate was maintained at 1 mL/min, and the injection volume was 10 μL. The detection was carried out at a wavelength of 254 nm.


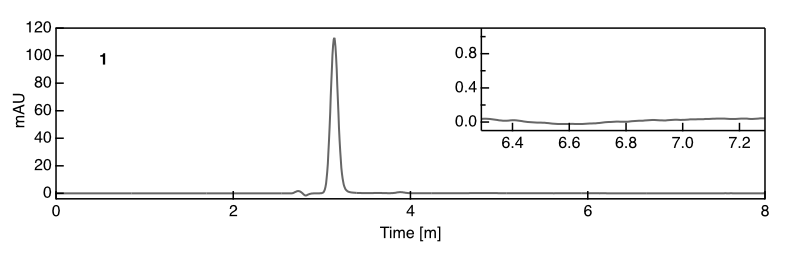


**Figure S14.** HPLC of reactant **1** dissolved in methanol.


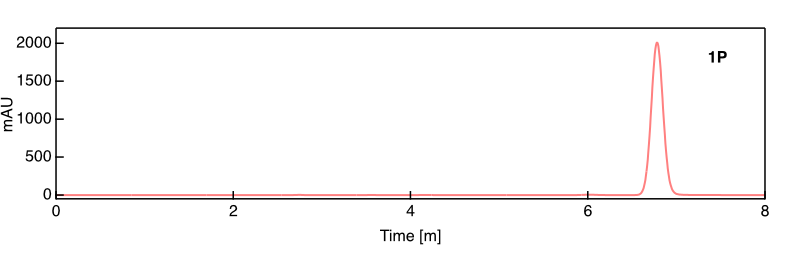


**Figure S15.** HPLC of product **1P** dissolved in methanol.


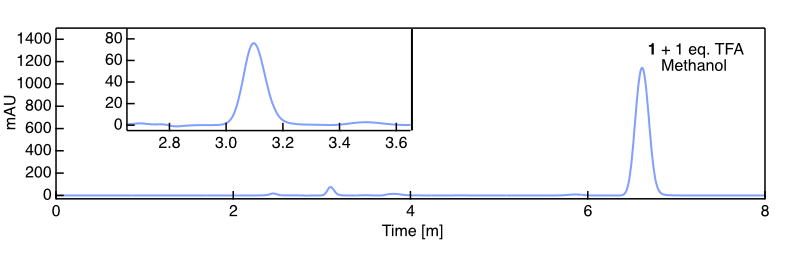


**Figure S16.** HPLC of 0.5 mg/mL reactant **1** with addition of 1 equiv of TFA in methanol. The ratio of the peak areas for **1** (462.50) and **1P** (12743.44) is 1:27.


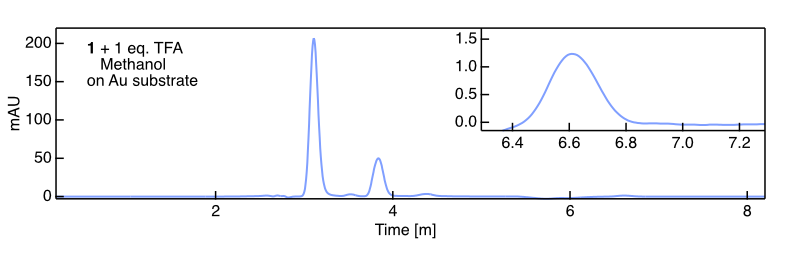


**Figure S17.** 10 μL of 1 mg/mL reactant **1** with addition of 1 equiv of TFA in methanol was placed on the Au substrate for 3 h. An additional 1 mL methanol was used to wash the mixture from the Au substrate. The collected mixed solution was analyzed by HPLC. The ratio of the peak areas for **1** (1142.77) and **1P** (12.74) is 90:1.


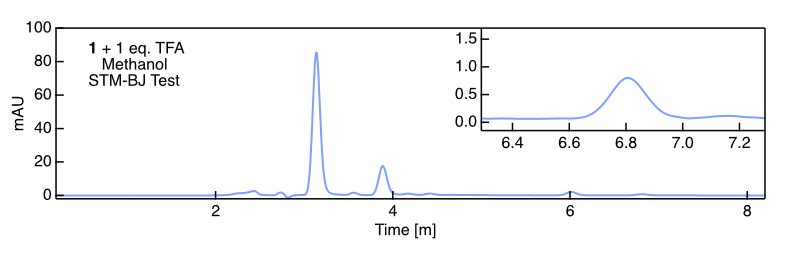


**Figure S18.** 10 μL of 1 mg/mL reactant **1** with addition of 1 equiv of TFA in methanol was placed on the Au substrate and reacted in the STM-BJ test for 3 h. An additional 1 mL methanol was used to wash the mixture from the Au substrate. The collected mixed solution was analyzed by HPLC. The ratio of the peak areas for **1** (527.79) and **1P** (6.41) is 82:1.


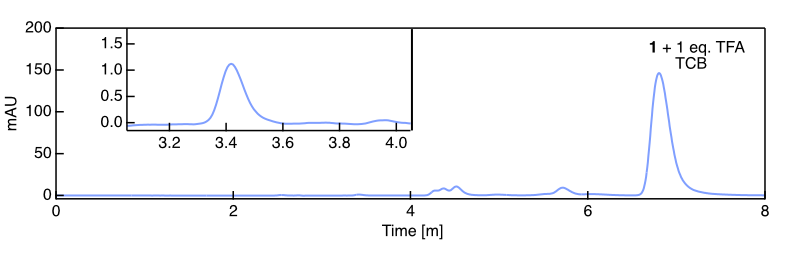


**Figure S19.** HPLC of 1 mM reactant **1** with addition of 1 equiv of TFA in TCB. The mixture is assumed to react with H_2_O upon its injection into the column for HPLC analysis.

In performing the HPLC experiments, the **1** + TFA solution will immediately react in a mobile phase that contains polar H_2_O solvent; thus, we cannot analyze the reaction products by using the HPLC method. Specifically, we emphasize that for Figure S19, the reaction will be exposed to H_2_O solvent upon injection into the HPLC system (mobile phase contains H_2_O), thus we cannot analyze the reaction in nonpolar TCB solvent in this method.

Method 4: Ultraviolet-visible spectroscopy (UV-vis)

We have performed UV-vis experiments for conforming the reaction products.


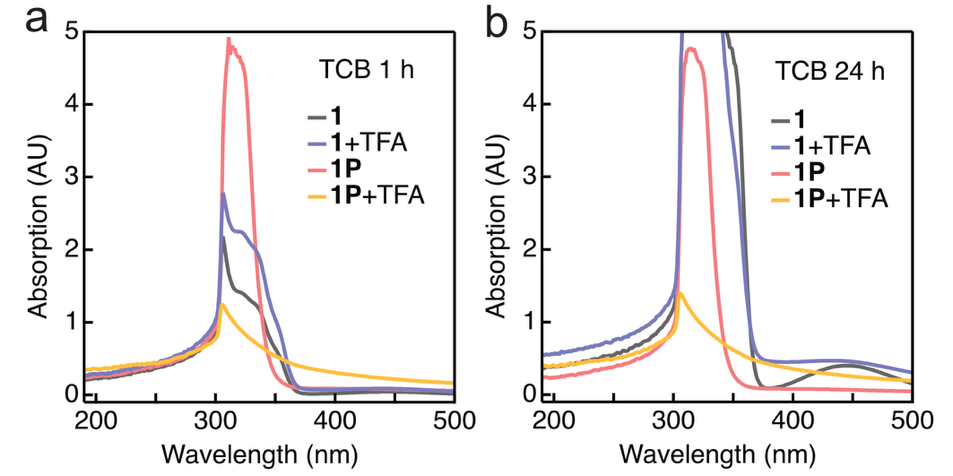


**Figure S20.** UV-vis spectra for **1**, **1** + 1 equiv of TFA, **1P**, and **1P** + 1 equiv of TFA, in the solvent of TCB, obtained after the reaction proceeded for (a) 1 h and (b) 24 h in air. The concentration for **1** and **1P** is 1 mM, which is the same as that used for the STM-BJ experiments.


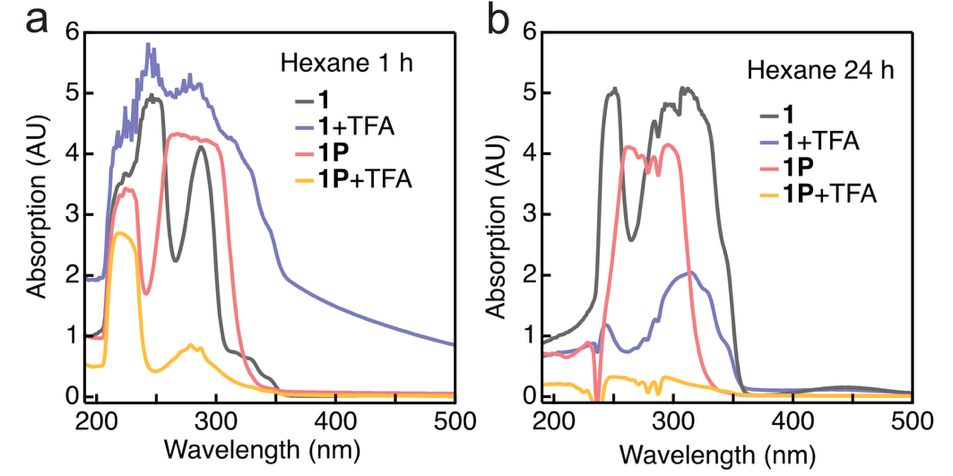


**Figure S21.** UV-vis spectra for **1**, **1** + 1 equiv of TFA, **1P**, and **1P** + 1 equiv of TFA, in the solvent of hexane, obtained after the reaction proceeded for (a) 1 h and (b) 24 h in air. The concentration for **1** and **1P** is 1 mM, which is the same as that used for the STM-BJ experiments.

We find that **1** and **1P** show similar absorption peaks, thus cannot be used for exclusively determining the reaction products. Specifically, we find that the absorption profiles of reactant **1** and product **1P** exhibit significant overlap, making it challenging to definitively verify the formation of **1P** using this method. In the UV-vis experiment which was performed 24 h after **1** was mixed with TFA, a distinct new absorption peak at 450 nm was observed (Figures S20b and S21b). This peak at 450 nm is attributed to the formation of azobenzene, likely resulting from the oxidation of **1** under room temperature and aerobic conditions.^7, 8^

**Section 5. NMR spectra**


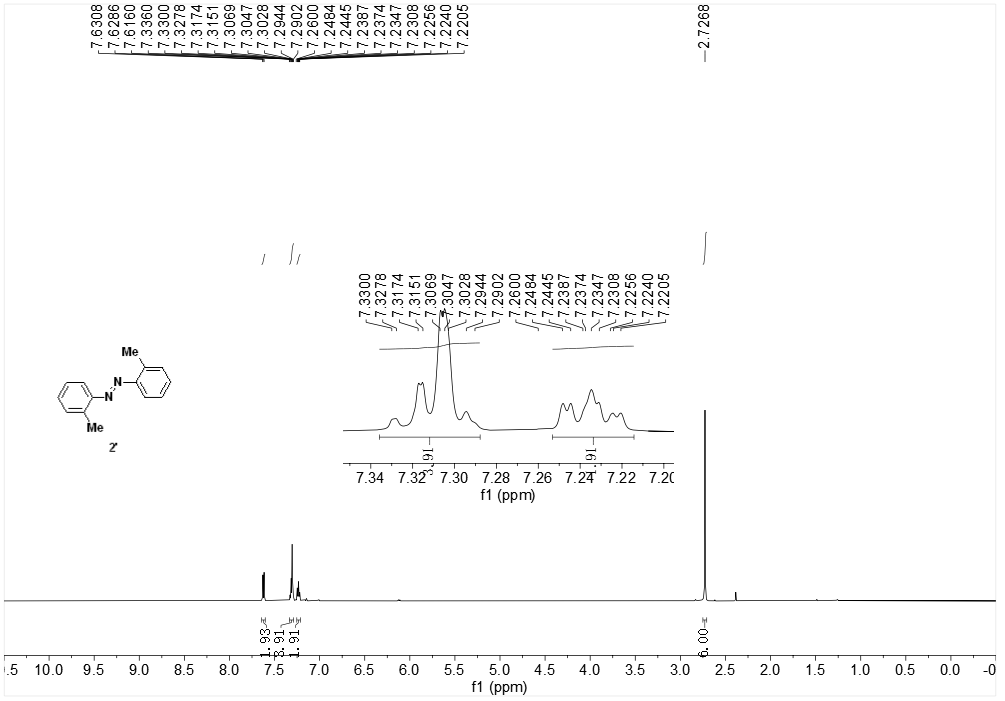


**Figure S22. ^1^**H NMR spectrum of compound **2’** (CDCl_3_, 25 ^o^C, 600 MHz).


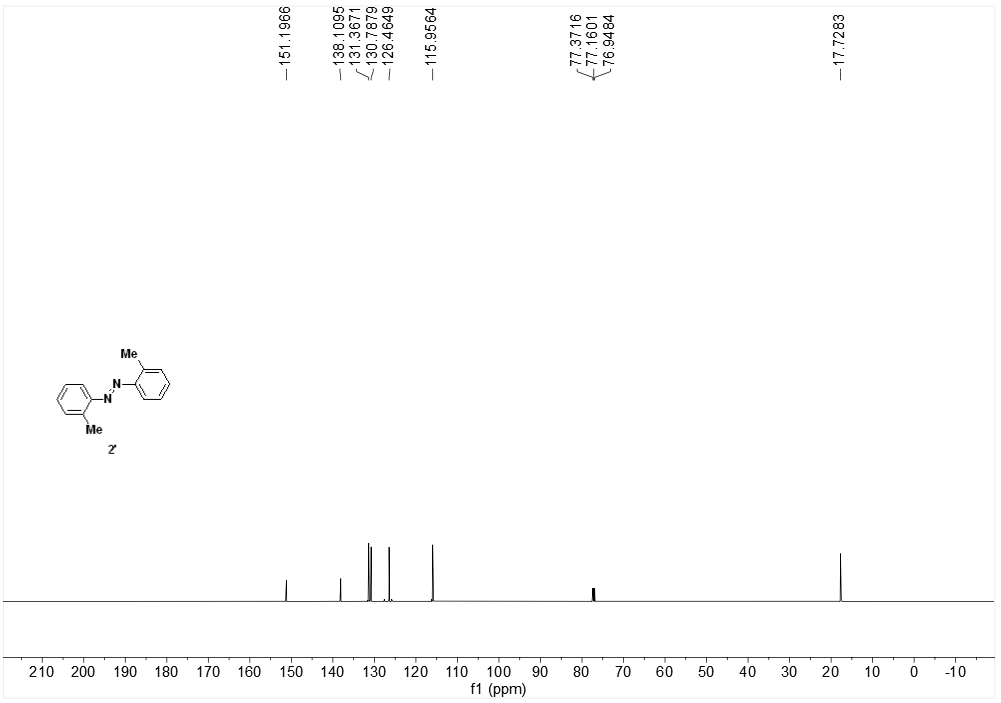


**Figure S23. ^13^**C NMR spectrum of compound **2’** (CDCl_3_, 25 ^o^C, 151 MHz).


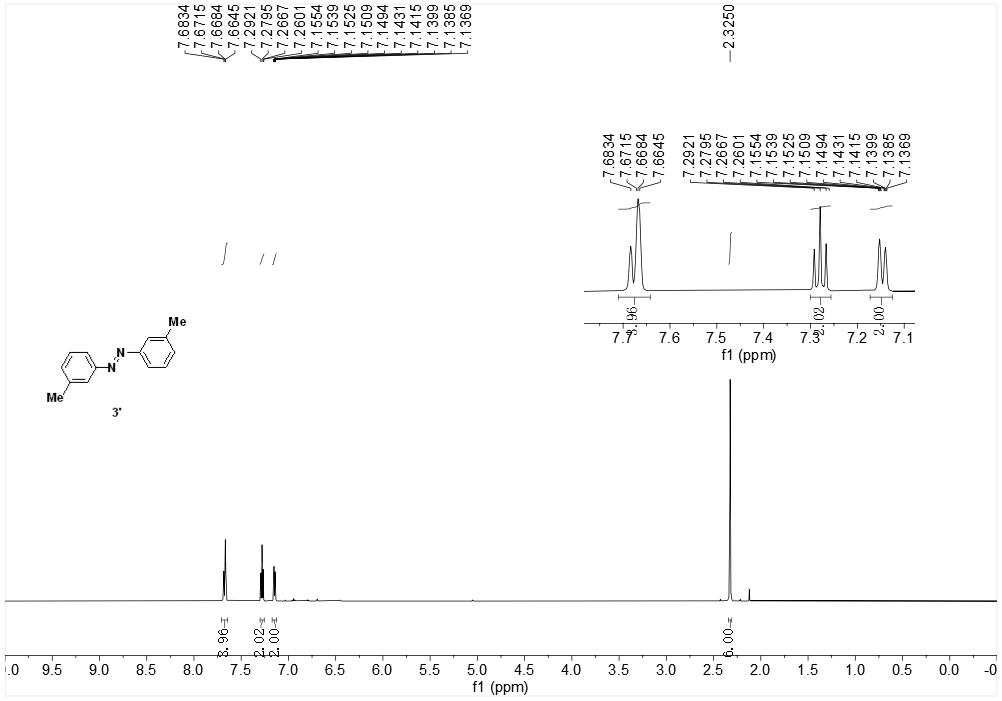


**Figure S24. ^1^**H NMR spectrum of compound **3’** (CDCl_3_, 25 ^o^C, 600 MHz).


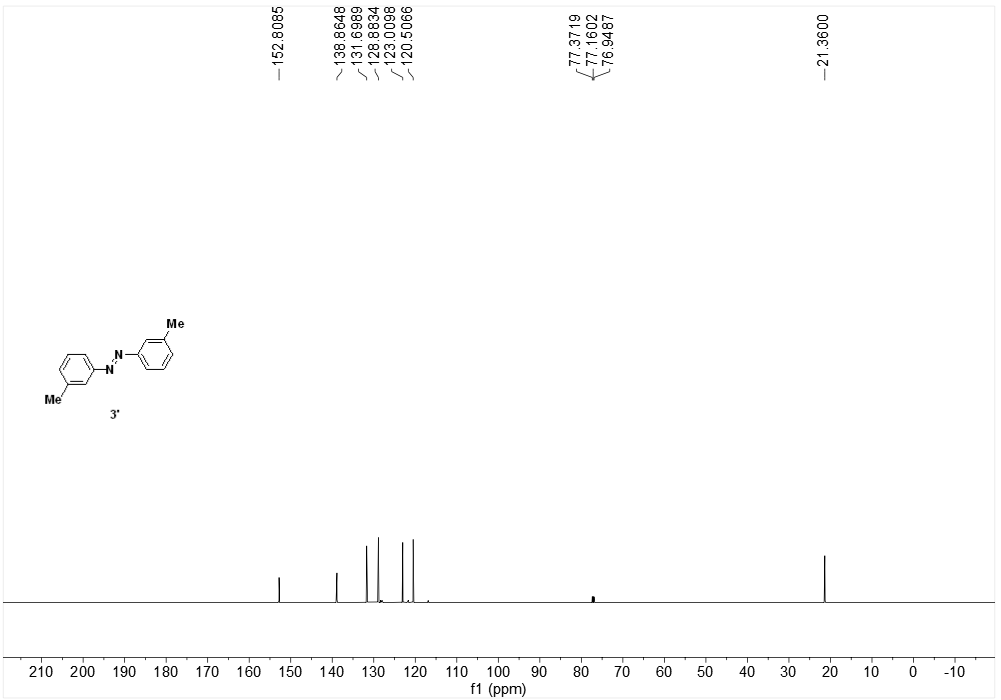


**Figure S25. ^13^**C NMR spectrum of compound **3’** (CDCl_3_, 25 ^o^C, 151 MHz).


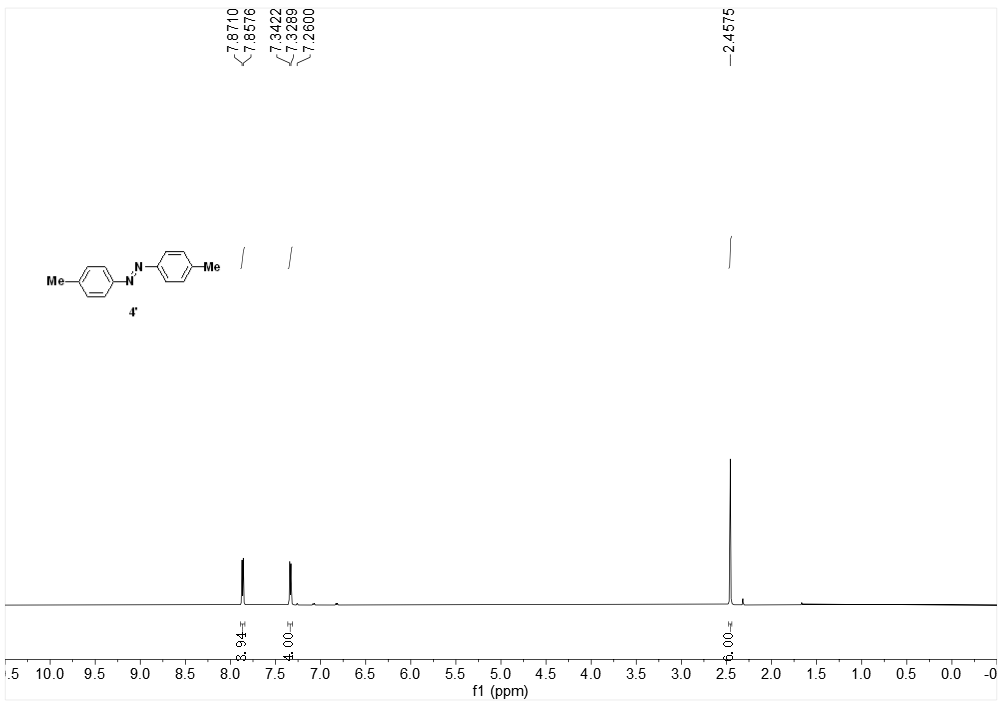


**Figure S26. ^1^**H NMR spectrum of compound **4’** (CDCl_3_, 25 ^o^C, 600 MHz).


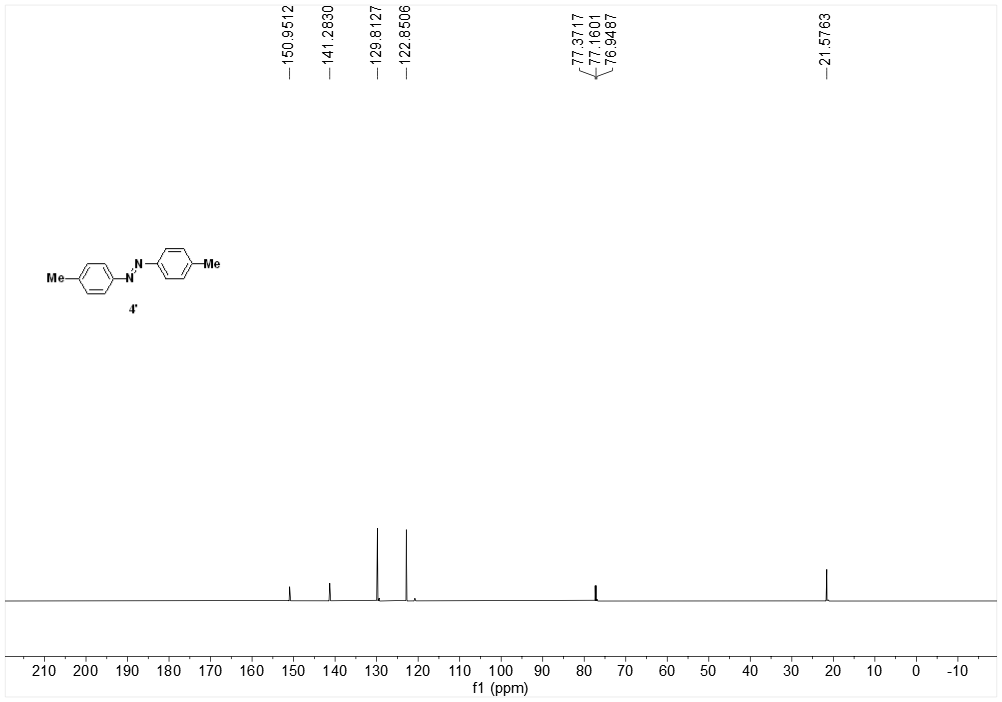


**Figure S27. ^13^**C NMR spectrum of compound **4’** (CDCl_3_, 25 ^o^C, 151 MHz).


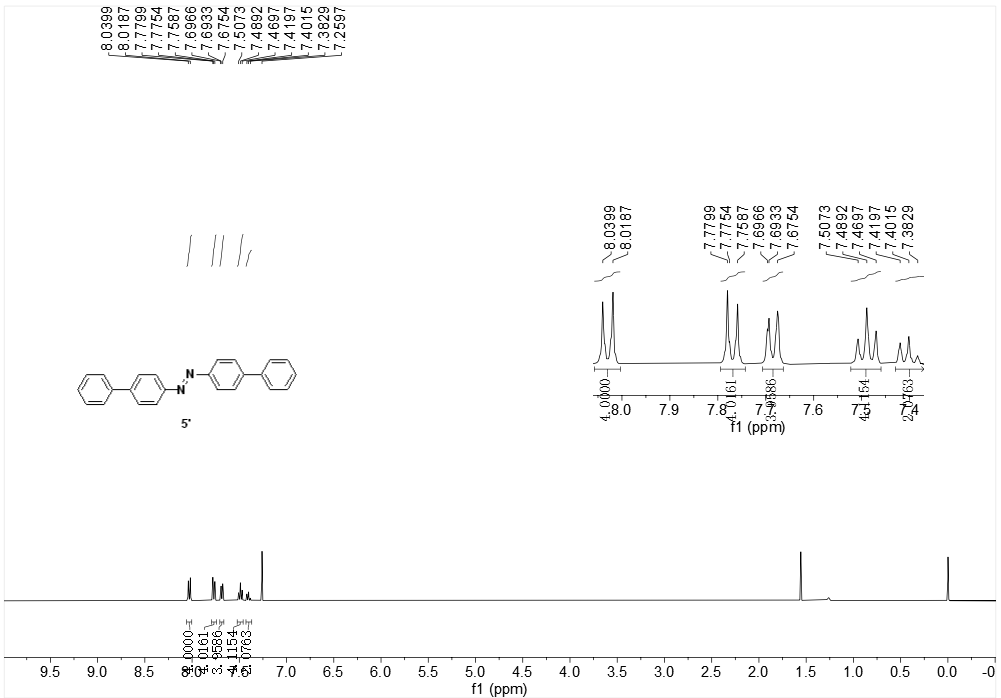


**Figure S28. ^1^**H NMR spectrum of compound **5’** (CDCl_3_, 25 ^o^C, 400 MHz).


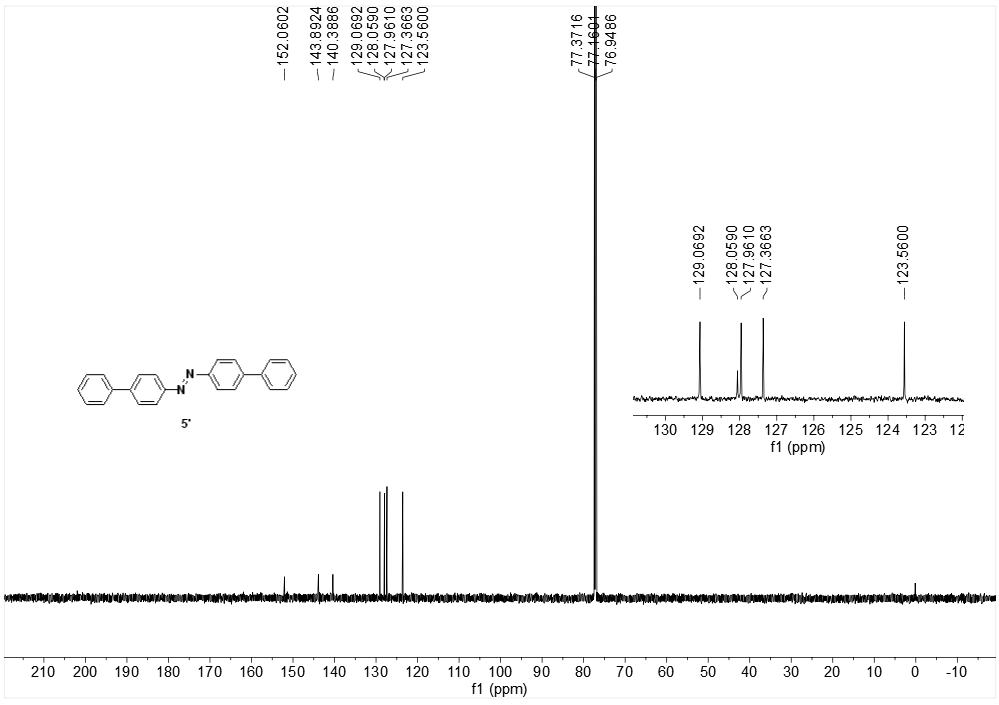


**Figure S29. ^13^**C NMR spectrum of compound **5’** (CDCl_3_, 25 ^o^C, 151 MHz).


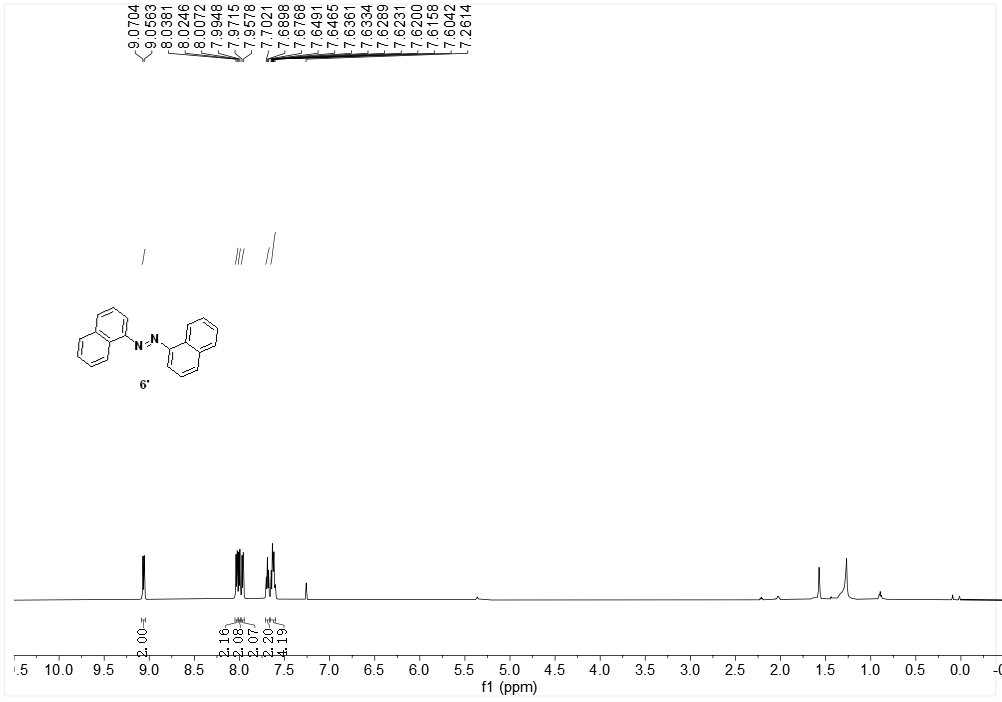
 **Figure S30. ^1^**H NMR spectrum of compound **6’** (CDCl_3_, 25 ^o^C, 600 MHz).


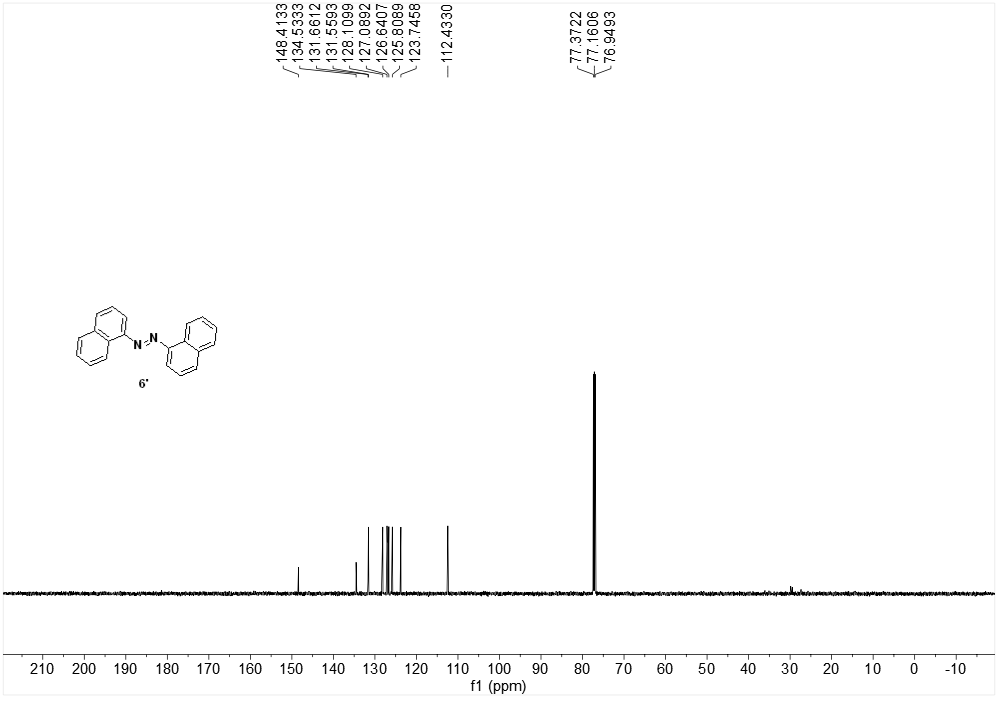


**Figure S31. ^13^**C NMR spectrum of compound **6’** (CDCl_3_, 25 ^o^C, 151 MHz).


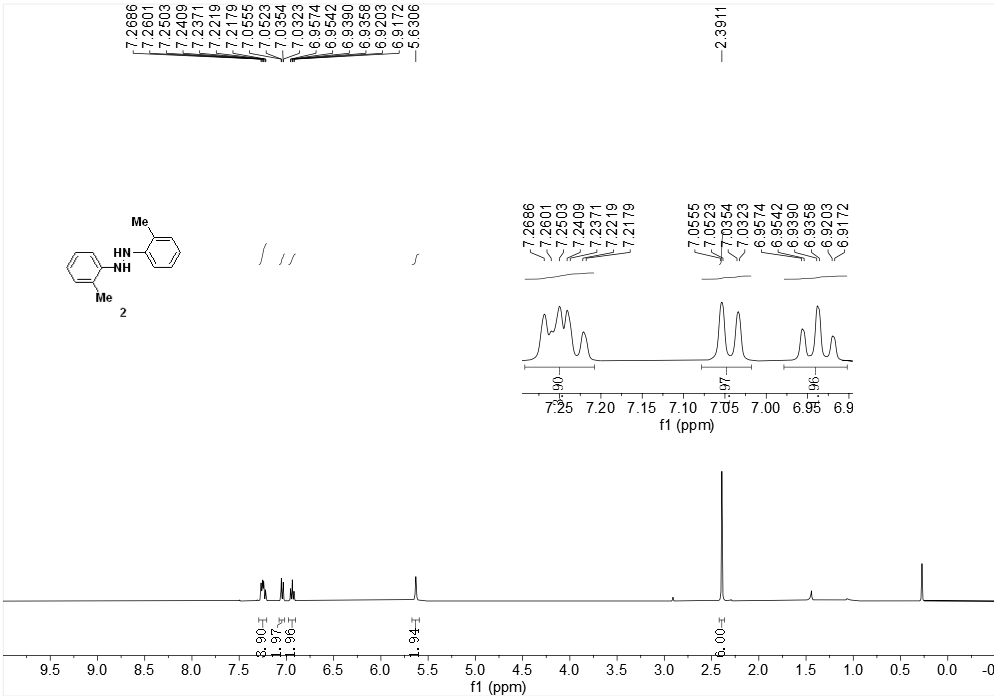


**Figure S32. ^1^**H NMR spectrum of compound **2** (CDCl_3_, 25 ^o^C, 400 MHz).


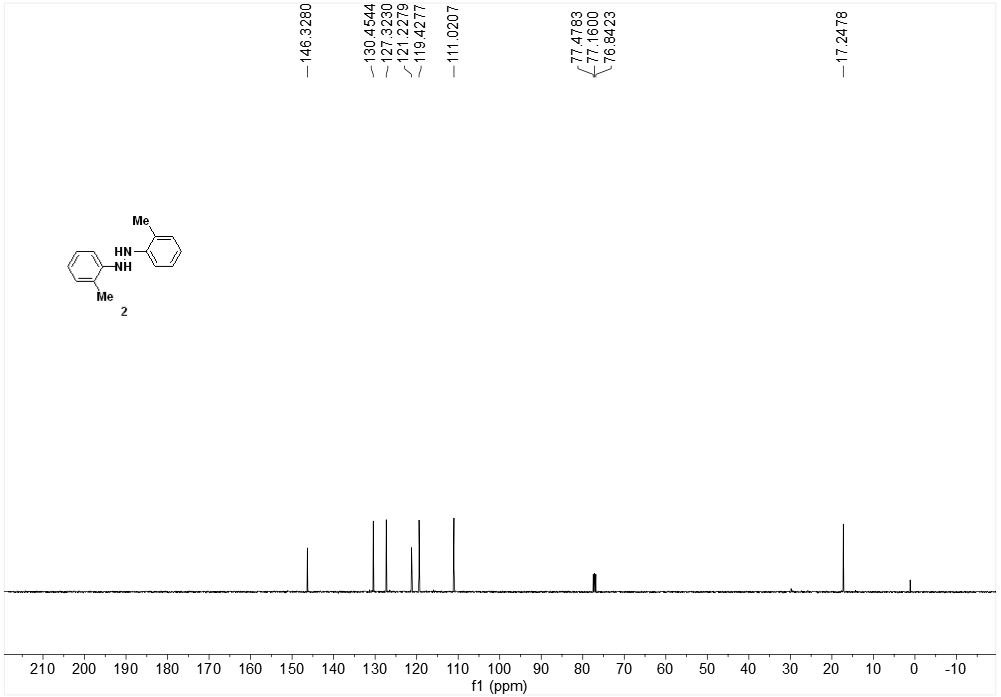


**Figure S33. ^13^**C NMR spectrum of compound **2** (CDCl_3_, 25 ^o^C, 101 MHz).


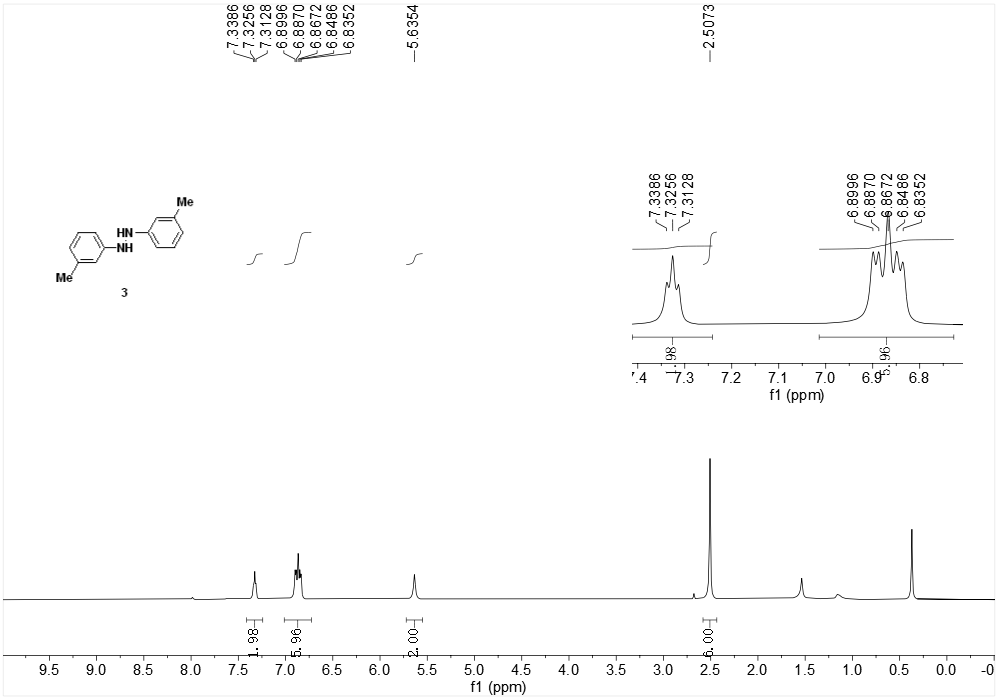


**Figure S34. ^1^**H NMR spectrum of compound **3** (CDCl_3_, 25 ^o^C, 600 MHz).


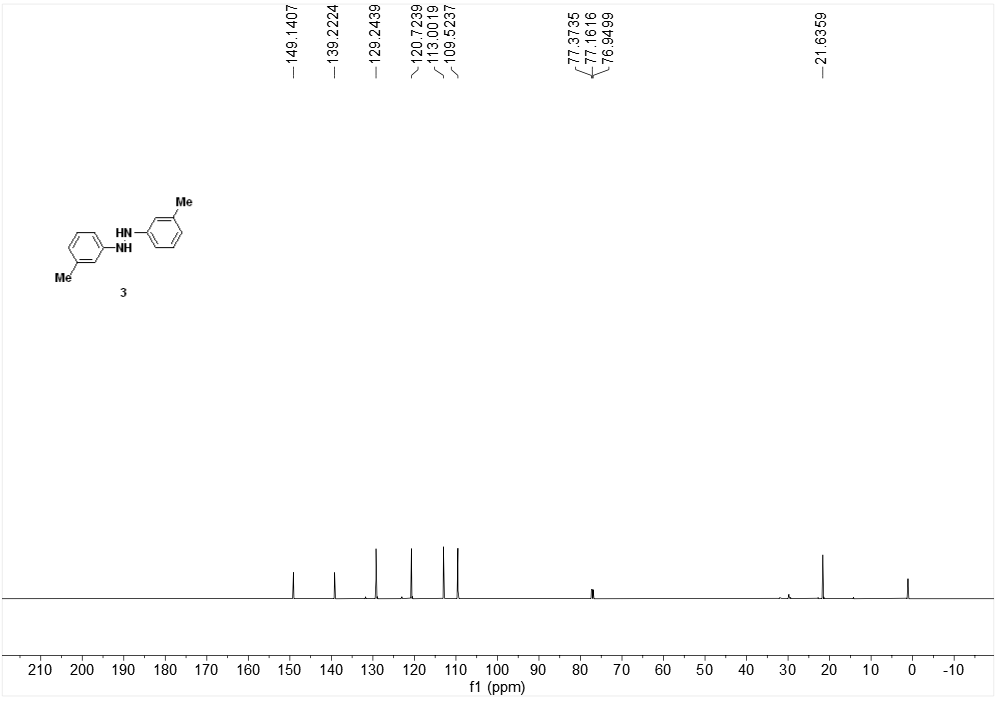


**Figure S35. ^13^**C NMR spectrum of compound **3** (CDCl_3_, 25 ^o^C, 151 MHz).


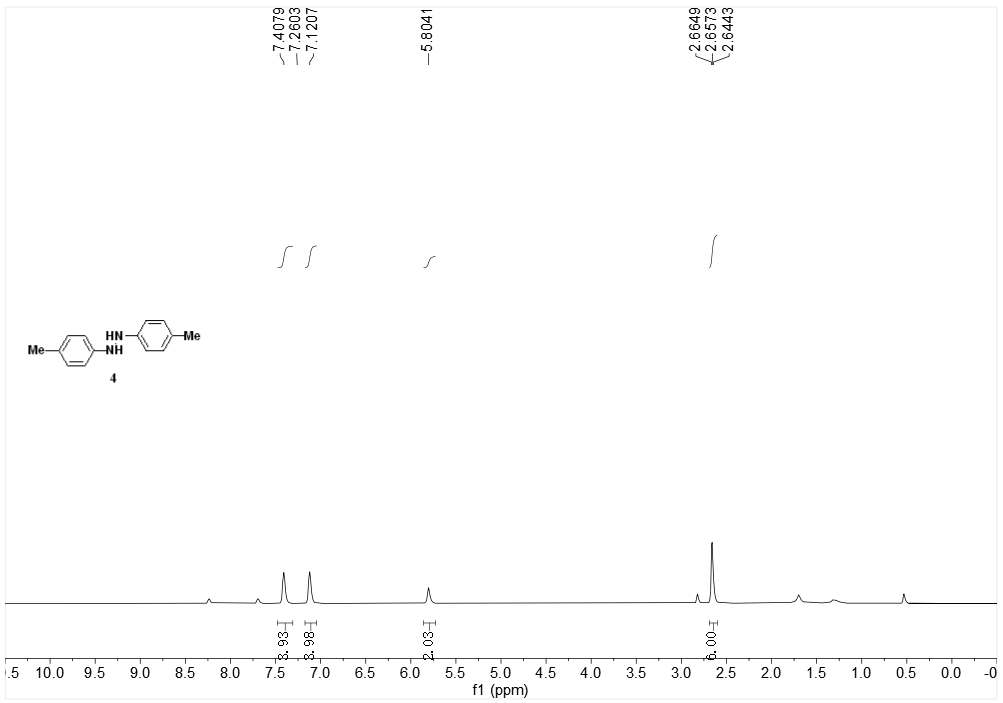


**Figure S36. ^1^**H NMR spectrum of compound **4** (CDCl_3_, 25 ^o^C, 600 MHz).


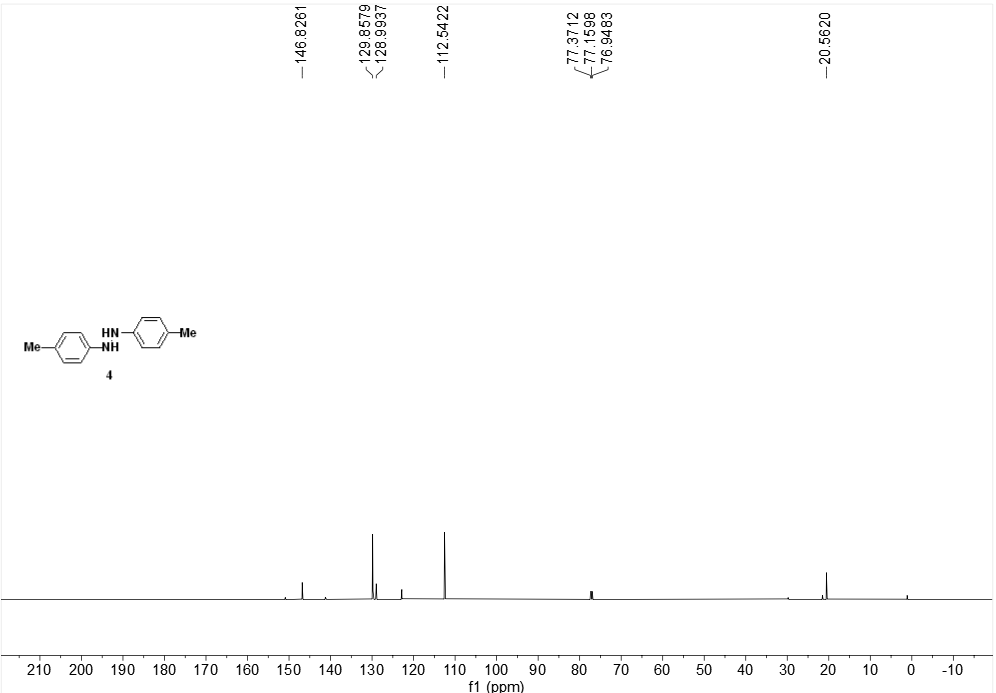


**Figure S37. ^13^**C NMR spectrum of compound **4** (CDCl_3_, 25 ^o^C, 151 MHz).


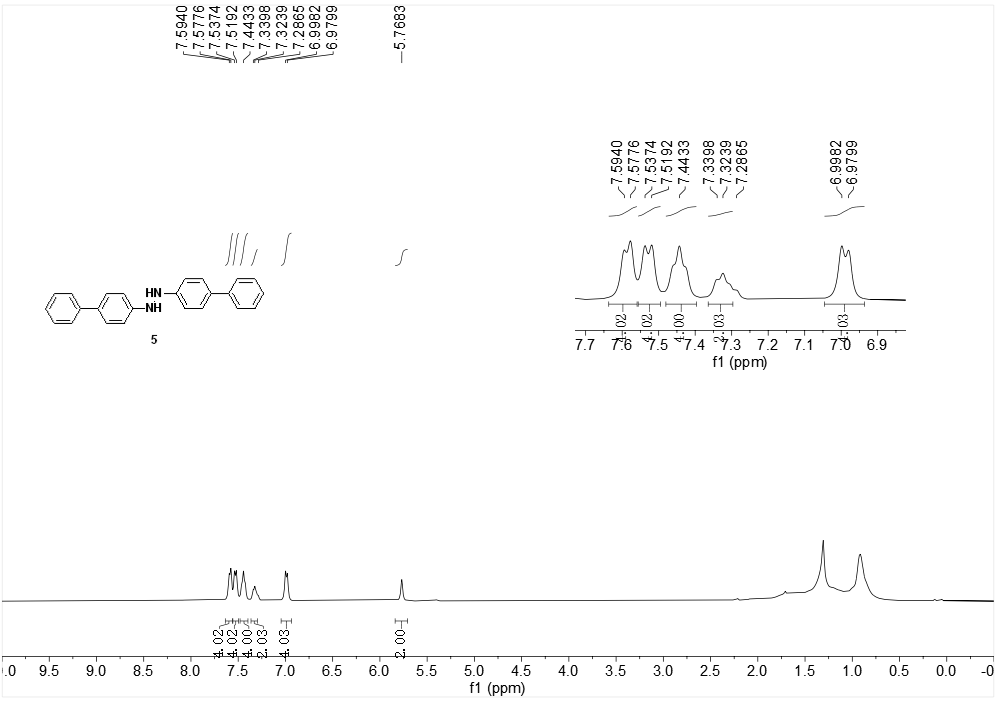


**Figure S38. ^1^**H NMR spectrum of compound **5** (CDCl_3_, 25 ^o^C, 400 MHz).


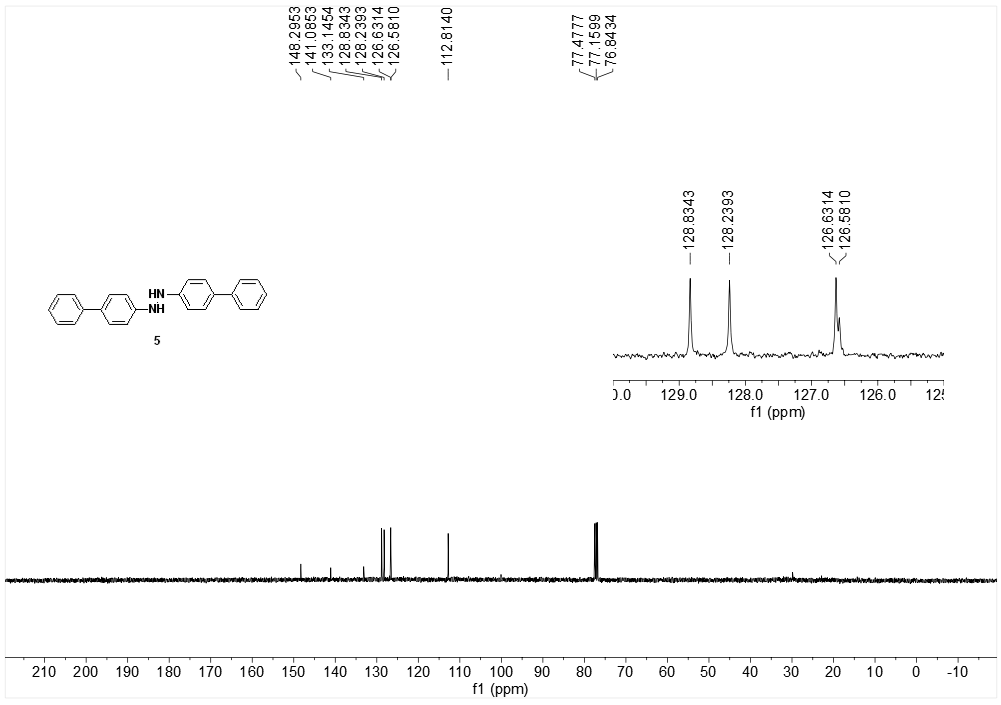


**Figure S39. ^13^**C NMR spectrum of compound **5** (CDCl_3_, 25 ^o^C, 101 MHz).


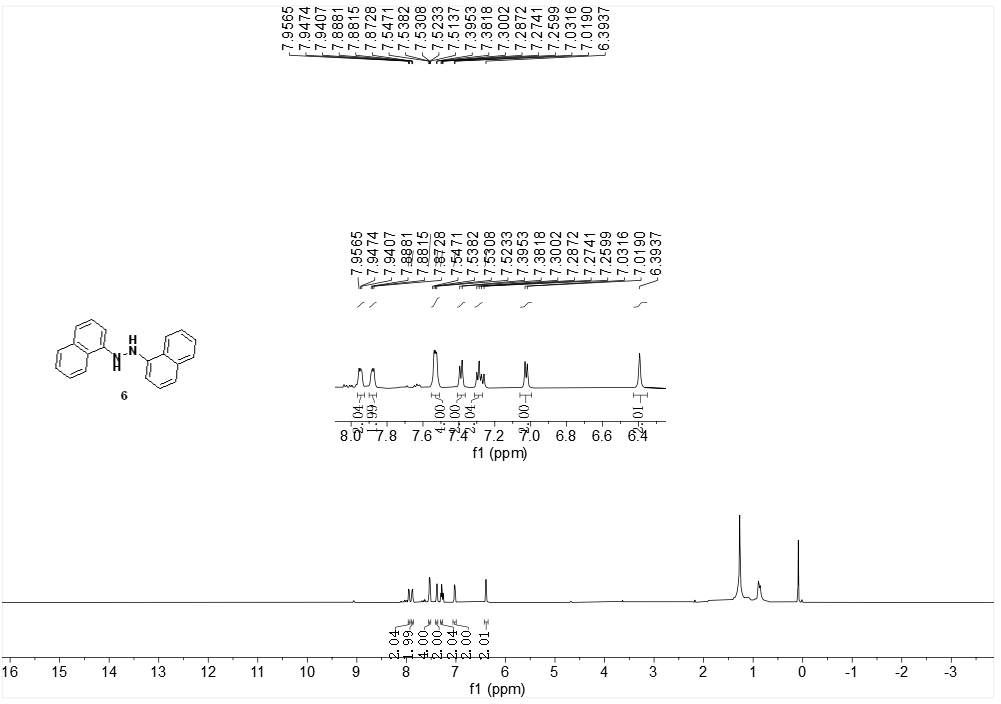


**Figure S40. ^1^**H NMR spectrum of compound **6** (CDCl_3_, 25 ^o^C, 600 MHz).


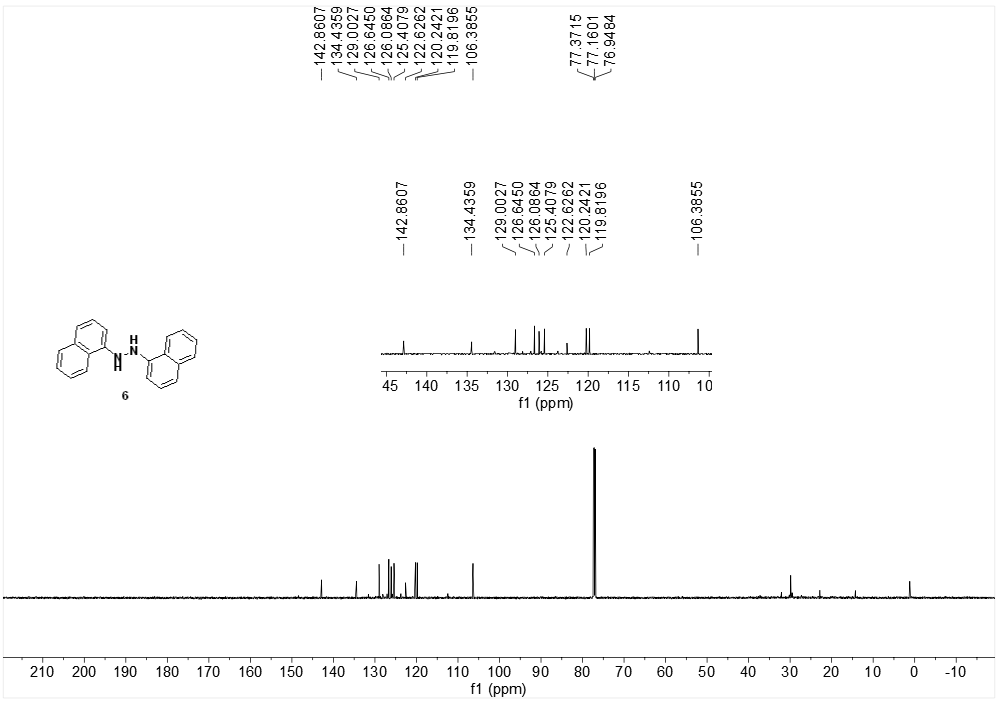


**Figure S41. ^13^**C NMR spectrum of compound **6** (CDCl_3_, 25 ^o^C, 151 MHz).


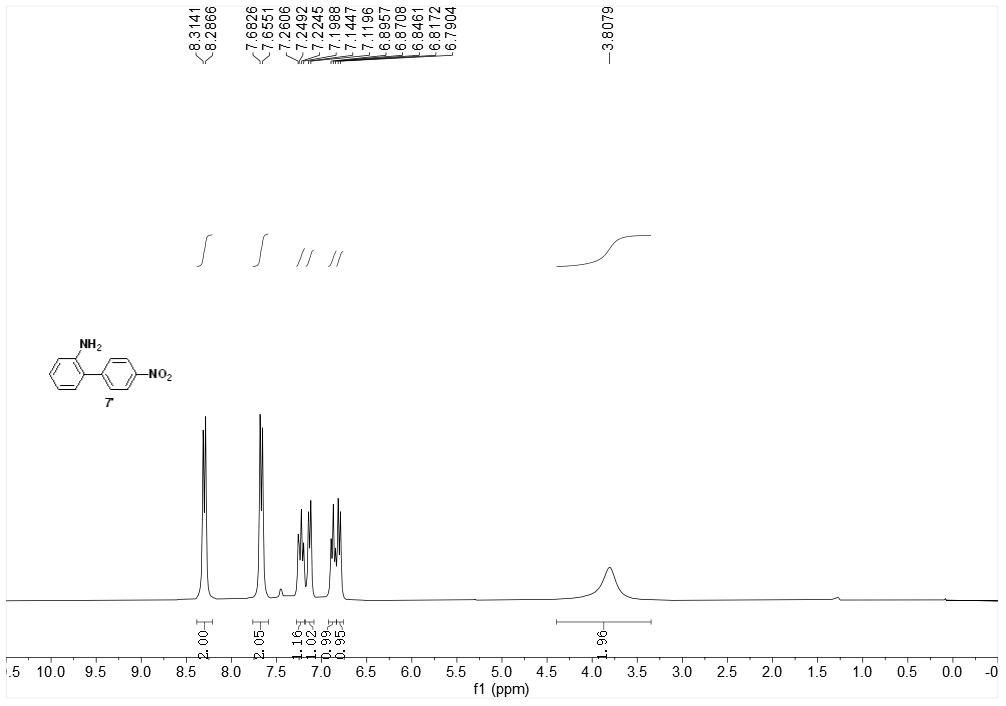
 **Figure S42. ^1^**H NMR spectrum of compound **7’** (CDCl_3_, 25 ^o^C, 300 MHz).


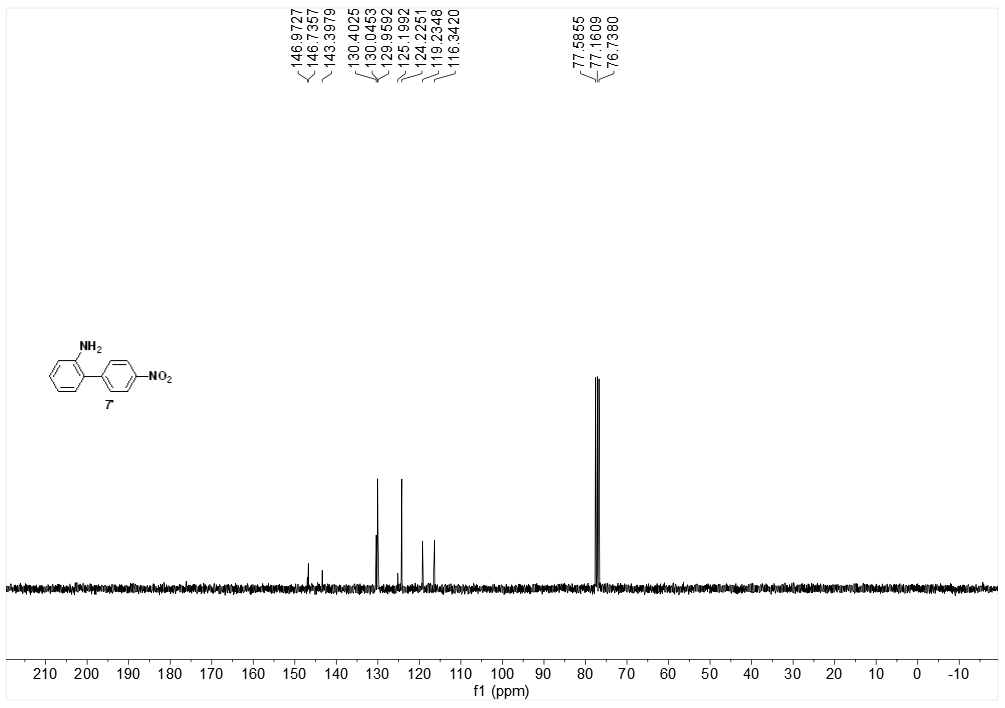
 **Figure S43. ^13^**C NMR spectrum of compound **7’** (CDCl_3_, 25 ^o^C, 75 MHz).


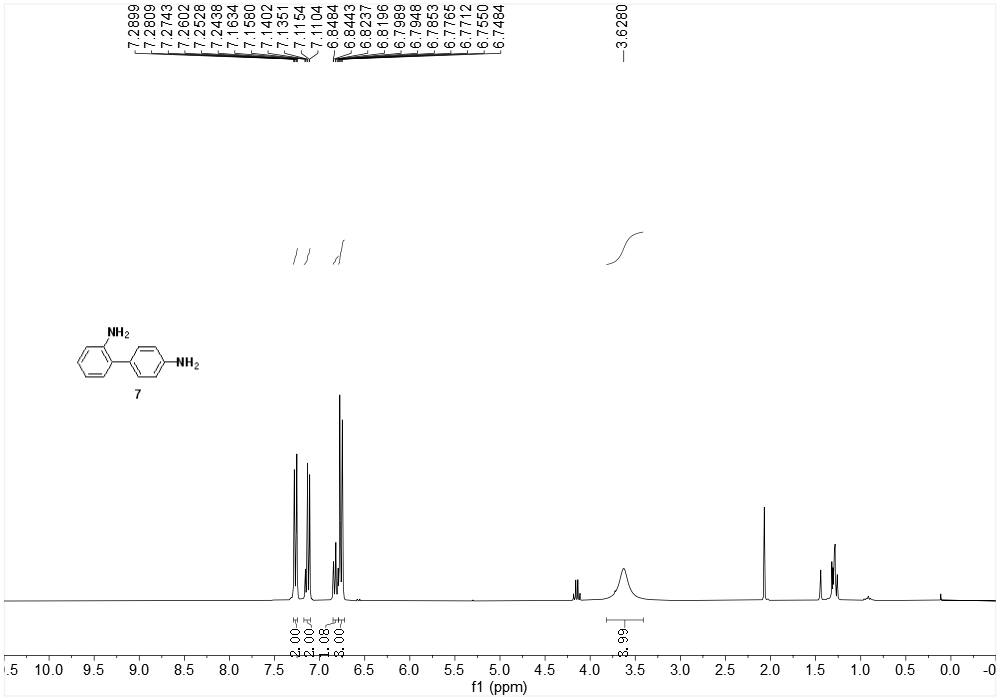
 **Figure S~~44~~. ^1^**H NMR spectrum of compound **7** (CDCl_3_, 25 ^o^C, 300 MHz).


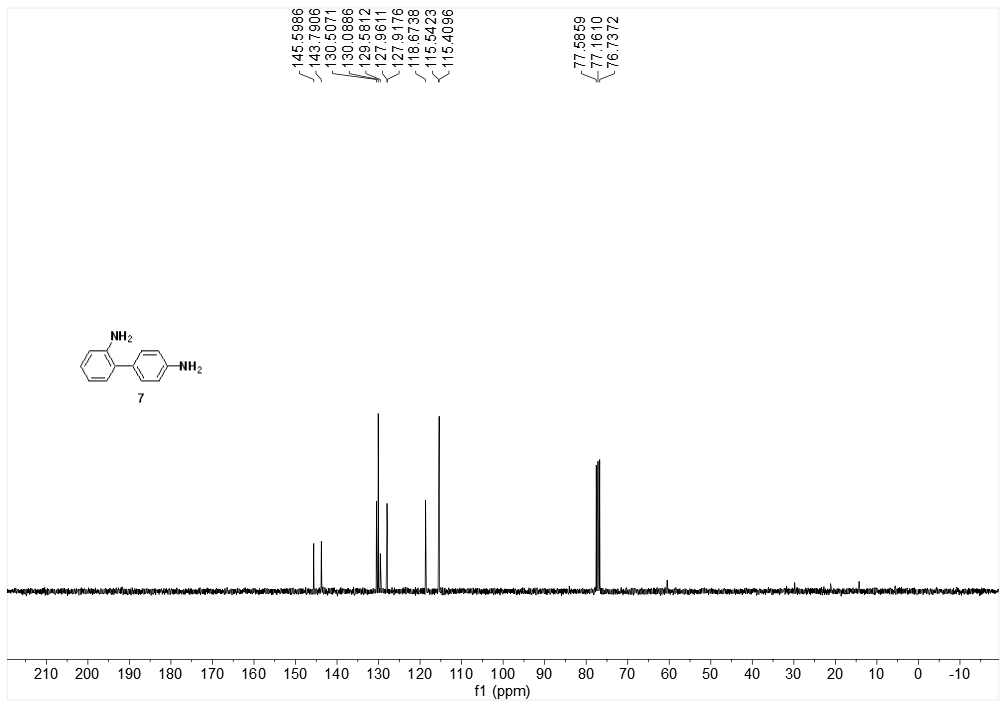


**Figure S45. ^13^**C NMR spectrum of compound **7** (CDCl_3_, 25 ^o^C, 75 MHz).


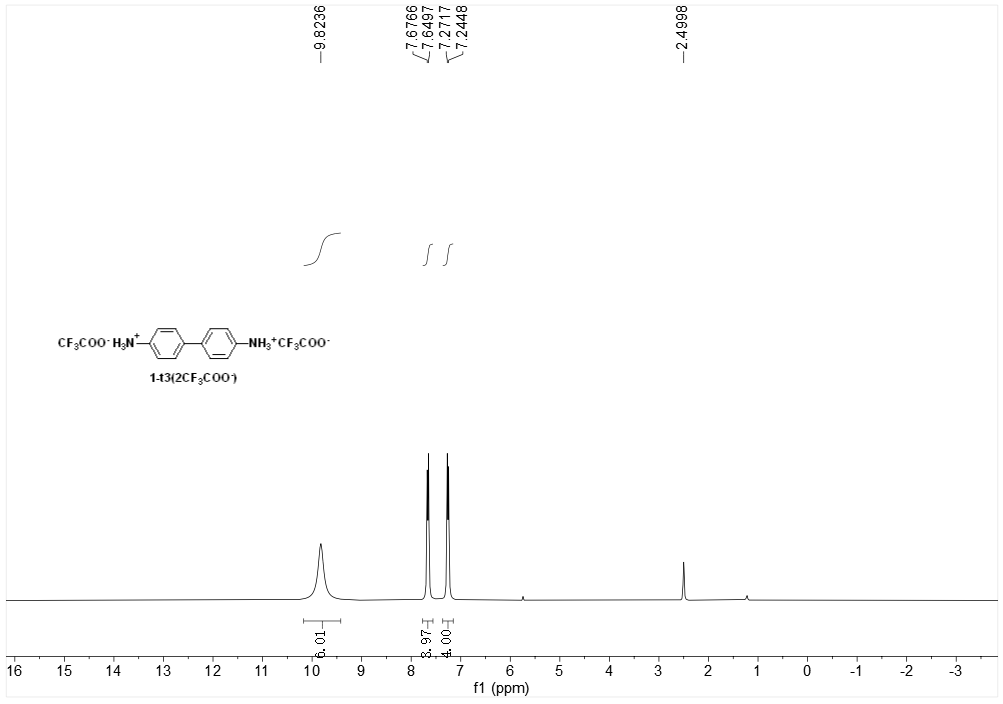
 **Figure S46. ^1^**H NMR spectrum of compound **1-t3** (2CF_3_COO^-^) (DMSO-*d6*, 25 ^o^C, 400 MHz).


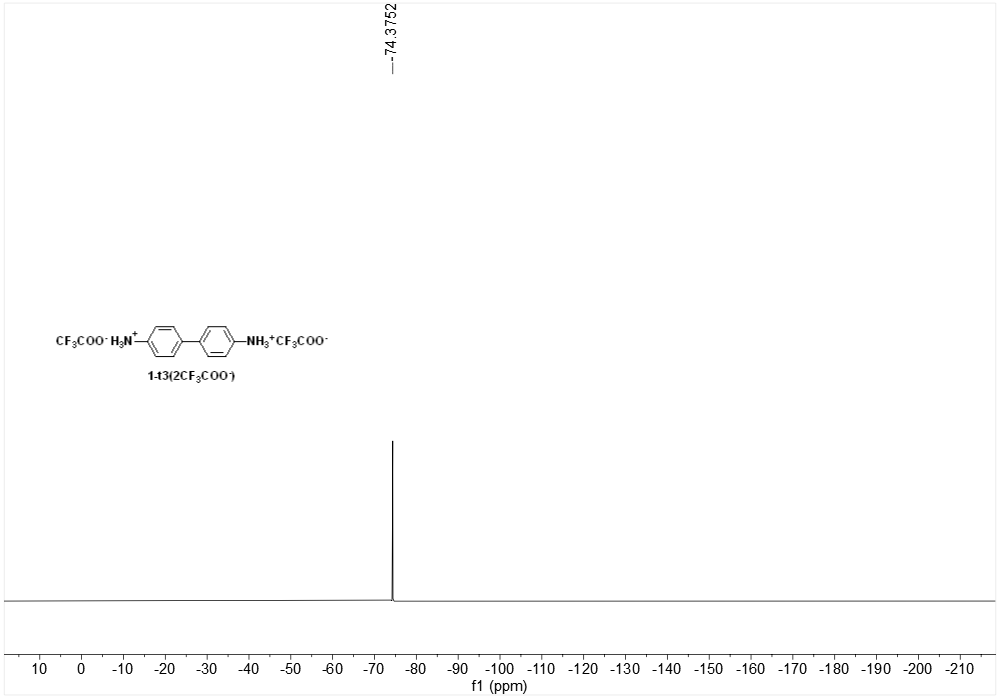


**Figure S47. ^19^**F NMR spectrum of compound **1-t3** (2CF_3_COO^-^) (DMSO-*d6*, 25 ^o^C, 400 MHz).


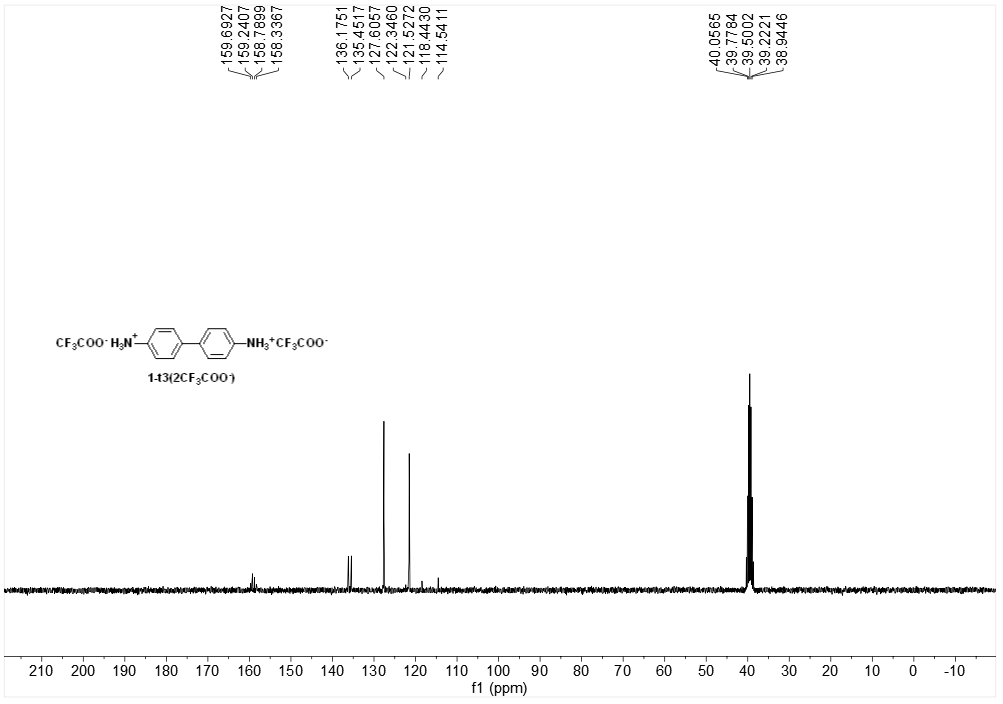


**Figure S48. ^13^**C NMR spectrum of compound **1-t3** (2CF_3_COO^-^) (DMSO-*d6*, 25 ^o^C, 101 MHz).

**Section 6. References**

(1) Mendenhall, G David; Smith, Peter AS. 2‐Nitrocarbazole: Carbazole, 2‐nitro. *Organic Syntheses* **2003**, *46*, 85-85.

(2) Bellamy, FD; Ou, K. Selective reduction of aromatic nitro compounds with stannous chloride in non acidic and non aqueous medium. *Tetrahedron Letters* **1984**, *25* (8), 839-842.

(3) Venkataraman, Latha; Klare, Jennifer E; Tam, Iris W; Nuckolls, Colin; Hybertsen, Mark S; Steigerwald, Michael L. Single-molecule circuits with well-defined molecular conductance. *Nano letters* **2006**, *6* (3), 458-462.

(4) Xu, Bingqian; Tao, Nongjian J. Measurement of single-molecule resistance by repeated formation of molecular junctions. *science* **2003**, *301* (5637), 1221-1223.

(5) Guo, Weiyi; Quainoo, Timothy; Liu, Zhen-Fei; Li, Haixing. Robust binding between secondary amines and Au electrodes. *Chemical Communications* **2024**, *60* (25), 3393-3396.

(6) Cox, RA; Buncel, E. The chemistry of the hydrazo, azo and azoxy groups. *John Wiley and Sons, New York* **1975**, 838.

(7) Yuan, Quan; Zhang, Yunfei; Chen, Yan; Wang, Ruowen; Du, Chaoling; Yasun, Emir; Tan, Weihong. Using silver nanowire antennas to enhance the conversion efficiency of photoresponsive DNA nanomotors. *Proceedings of the National Academy of Sciences* **2011**, *108* (23), 9331-9336.

(8) Jiao, Qing; Yi, Zhuo; Chen, Yongming; Xi, Fu. Synthesis and encapsulation properties of dendronized polymer with Fréchet‐type dendrons peripherally decorated by carboxylic acid functionalization. *Journal of Polymer Science Part A: Polymer Chemistry* **2008**, *46* (13), 4564-4574.
